# Finite element analysis of the interaction between high-compliant balloon catheters and non-cylindrical vessel structures: towards tactile sensing balloon catheters

**DOI:** 10.1007/s10237-023-01749-8

**Published:** 2023-08-13

**Authors:** Ashish Bhave, Benjamin Sittkus, Gerald Urban, Ulrich Mescheder, Knut Möller

**Affiliations:** 1 Institute of Technical Medicine (ITeM), Furtwangen University, 78054 Villingen-Schwenningen, Germany; 2 Department of Microsystems Engineering, IMTEK, University of Freiburg, 79110 Freiburg, Germany; 3 Institute for Microsystems Technology (iMST), Furtwangen University, 78120 Furtwangen, Germany; 4 Associated to the Faculty of Engineering, University of Freiburg, 79110 Freiburg, Germany; 5 Department of Mechanical Engineering, University of Canterbury, Christchurch, New Zealand

**Keywords:** Finite element analysis, Balloon catheter, Tactile sensing, Contact simulation, Vessel analysis, Reverse identification

## Abstract

Aiming for sensing balloon catheters which are able to provide intraoperative information of the vessel stiffness and shape, the present study uses finite element analysis (FEA) to evaluate the interaction between high-compliant elastomer balloon catheters with the inner wall of a non-cylindrical-shaped lumen structure. The contact simulations are based on 3D models with varying balloon thicknesses and varying tissue geometries to analyse the resulting balloon and tissue deformation as well as the inflation pressure dependent contact area. The wrinkled tissue structure is modelled by utilizing a two-layer fibre-based Holzapfel-Gasser-Ogden constitutive model and the model parameters are adapted based on available biomechanical data for human urethral vessel samples. The balloon catheter structure is implemented as a high-compliant hyper-elastic silicone material (based on polydimethylsiloxane (PDMS)) with a varying catheter wall thickness between 0.5 and 2.5 µm. Two control parameters are introduced to describe the balloon shape adaption in reaction to a wrinkled vessel wall during the inflation process. Basic semi-quantitative relations are revealed depending on the evolving balloon deformation and contact surface. Based on these relations some general design guidelines for balloon-based sensor catheters are presented. The results of the conducted in-silico study reveal some general interdependencies with respect to the compliance ratio between balloon and tissue and also in respect of the tissue aspect ratio. Further they support the proposed concept of high-compliant balloon catheters equipped for tactile sensing as diagnosis approach in urology and angioplasty.

**Supplementary Information:**

The online version contains supplementary material available at 10.1007/s10237-023-01749-8.

## Introduction

**Fig. 1:**
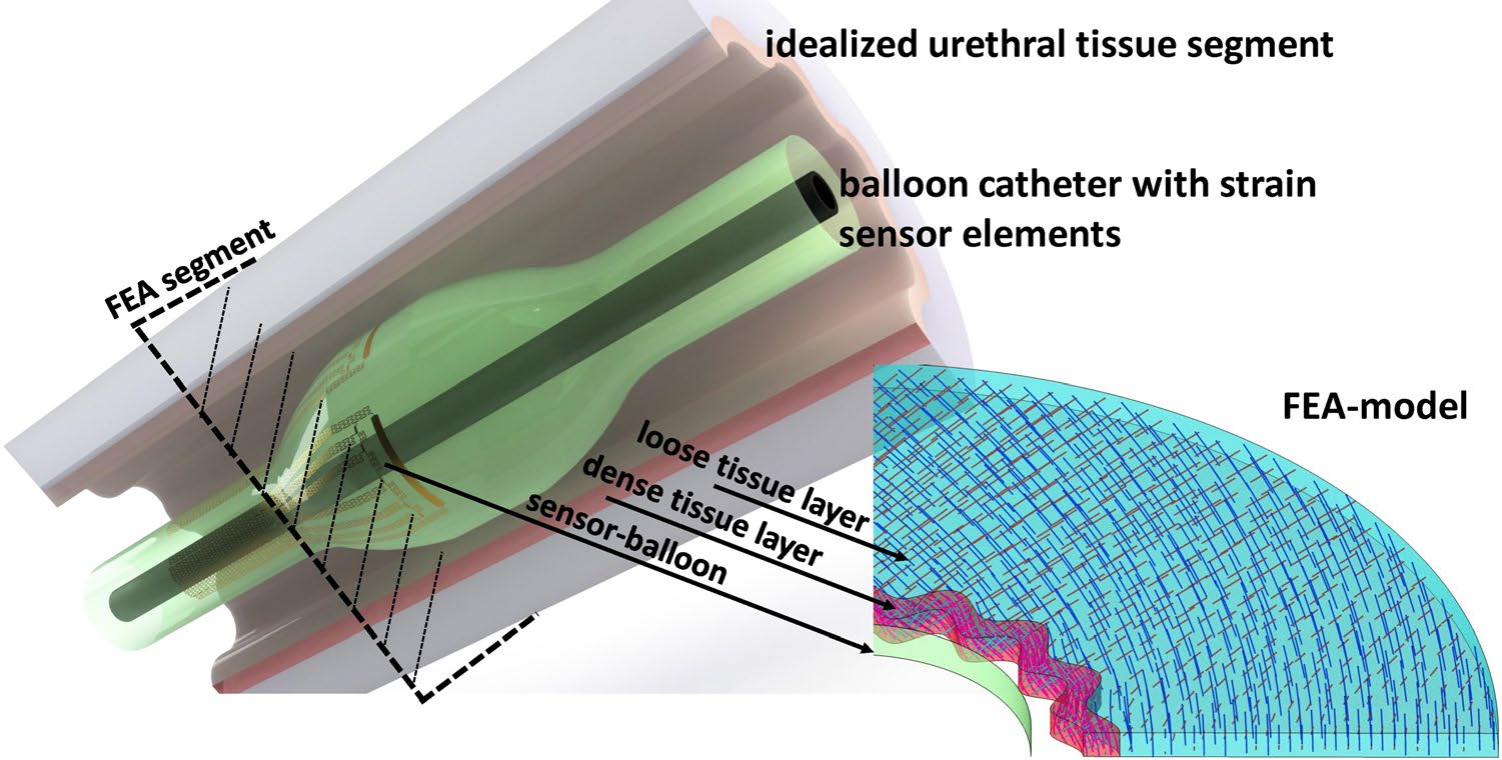
Schematic showing the motivation of the conducted FEA study

In minimally invasive procedures a surgeon is relying on image-based information of the intervention site, while his palpatory sense is unavailable. New medical instruments may “sense” relevant mechanical in situ tissue data from the site of operation. Combined real-time analysis of these obtained datasets with prior knowledge about a patient paves a promising route to an individual treatment of patients, e.g. in modern procedures of vessel interventions. For an effective individual treatment, quantitative data and real-time interpretation based on the patient’s status is mandatory. The present FEA study is motivated by the idea of designing functionalized balloon catheter devices with integrated highly stretchable strain sensors which provide the circumferential expansion at defined areas of the balloon structure (Fig. 1). With a suitable placement of sensor structures and appropriate reverse modelling a quantitative assessment of the endothelium shape and stiffness inside the targeted vessel structure can be obtained. Ultimately this aims for an intraoperative mapping of the vessel endothelium based on intraluminal mechanical tissue analysis, eventually enhanced by intraoperative standard imaging.

Currently catheter procedures target the revascularization of diseased vessel structures, e.g. coronary arteries (percutaneous coronary intervention, PCI)(Claessen et al. 2017) or peripheral limb arteries (percutaneous transluminal angioplasty, PTA)(Rogers und Laird 2007) or the restoration of urethral functionality by dilating strictures (Vyas et al. 2013; Chhabra et al. 2016), or drainage of the urinary bladder for diagnostic or therapeutic purposes (Lawrence und Turner 2005). During the latter procedures the functionality of the inserted balloon is rather simple and provides fixation of the catheter at the urethral transition to the bladder. Within balloon angioplasty or the balloon dilatation in urethral strictures, the balloon functionality resembles an actuator, dilating either the vessel structure itself or is used for the expansion of a stent to stabilize the expanded lumen. For the drainage of the bladder, the interaction of the catheter with the surrounding tissue must be avoided during the expansion (Baker et al. 2013). Generally, the cylindrical lumen shape (axial cut) of the expanding balloon remains (mostly) unchanged as it fulfils an actuator purpose (Helou et al. 2021; Auer et al. 2008, 2010; Vyas et al. 2013; Chhabra et al. 2016).

In respect of diagnosis of vessel properties, i.e. for intravascular measurements and 3D reconstruction of vessel geometries, established methods are the 3D IVUS (intravascular ultrasound) technique, a combination of IVUS with biplane angiography and the 3D QCA (Quantitative Coronary Angiography), which uses two angiographic planes. Additionally, 3D OCT (Optical Coherence Tomography) combining OCT with biplane angiography showed also comparable results to the prior named methods (Toutouzas et al. 2015). These methods mainly contribute to the field of coronary artery imaging for a better understanding of the hemodynamics associated with coronary artery diseases (Toutouzas et al. 2015), mostly in the framework of experimental studies. For accurate intravascular diameter and cross-sectional area measurements the balloon catheter-based Metricath® system (Neovasc Inc., formerly Angiometrx Inc., Canada) showed similar results as IVUS (van der Giessen et al. 2002, 2005). Schmidt et al. (Schmidt et al. 2018) investigated the compliance of surrounding tubes by using a pressure–volume control unit combined with the Metricath® balloon catheter. An in vitro study to assess the contact pressure applied to the vessel wall during balloon dilatation was conducted by Moriwaki et al. introducing a film-type pressure sensor between the dilating balloon and a cylindrical vessel model (Moriwaki et al. 2018), while in another work of that group the pressure distribution in contact with a plaque vessel model was evaluated (Moriwaki et al. 2018). Further, two independent works from 2011 and 2018 evaluate the possibility for compliance investigation of surrounding tissue with a camera-tracked balloon expansion (Ishii et al. 2011; Saito et al. 2019).

As stretchable sensor technologies have become a viable research topic during the last decade (Matsuhisa et al. 2019; Wu et al. 2020), the successes within this area facilitate the development and design of multifunctional sensing balloon catheters as proposed and demonstrated by Kim and co-workers back in 2011 and more recently by Han et al. 2020 (Kim et al. 2011; Han et al. 2020). Compliant stretchable strain sensors enable strain analysis of surfaces even under large mechanical deformations (Wu et al. 2020). Consequently, such sensors permit a detailed analysis of the pressure-dependent deformation along a balloon-catheter surface.

Several FEA studies related to the balloon inflation in angioplasty interventions can be found in literature (Oh et al. 1994; Gajewski et al. 2019; Gasser und Holzapfel 2007; Takashima et al. 2007; Helou et al. 2021; Helou et al. 2020; Martin und Boyle 2013; Liang et al. 2005; Geith et al. 2019; Kiousis et al. 2009; Wang et al. 2006; Wu et al. 2007). As stenting is common in PCI and PTA most of them are focussing on stent systems (Liang et al. 2005; Martin und Boyle 2013; Geith et al. 2019; Kiousis et al. 2009; Takashima et al. 2007; Wu et al. 2007). Some concentrate on the stent–tissue interaction (Takashima et al. 2007; Liang et al. 2005; Wu et al. 2007) or on the mechanics during the expansion of the balloon-stent system itself (Martin und Boyle 2013; Kiousis et al. 2009; Wang et al. 2006), or deal with the balloon–tissue interaction during balloon angioplasty (Helou et al. 2020, 2021; Oh et al. 1994; Gasser und Holzapfel 2007), or intra-aortic balloon occlusion (Gajewski et al. 2019). The only study known to the authors of this article which investigates a balloon expansion related to the urogenital tract is so far a FEA study on the pressure-dependent inflation behaviour of a Foley catheter (Shuib et al. 2007). Another FEA study with relation to the urogenital tract can be found in a more recent work of Natali et al. that evaluates the interaction between an artificial urinary sphincter on the urethral duct (Natali et al. 2020). A constitutive formulation and structural modelling of the urethral duct is provided in a preceding work of these authors (Natali et al. 2017).

We present investigations for a tactile assessment of the surrounding vessel compliance and shape. The tissue interaction and shape deformation of the balloon during the inflation process is the governing factor determining the diagnostic capabilities of such balloon sensors. Consequently, the boundary conditions of the aforementioned angioplasty-related studies are not suitable, as the high balloon stiffness preserves the cylindrical balloon shape and the pressure-dependent expansion rate during the contact with much more compliant vessel structures. In contrast, the current study evaluates the interaction of high-compliant elastomer-based balloons in non-cylindrical structures, where the balloon compliance is in the same range as the compliance of the surrounding tissue. This aims for diagnostic balloon catheters which can detect irregularly shaped healthy, i.e. non-fibrosed vessel structures and therefore might even provide data for early detection of diseased tissue.

As the common approach in angioplasty-related studies is based on idealized cylindrical vessel structures, and deviating shapes are related to a stiff-calcified lesion (e.g. (Helou et al. 2021)), in this study a fibre-based tissue model (Holzapfel et al. 2000) is adapted according to available biomechanical data of the urethra. However, as this study strives to reveal general dependencies for the balloon interaction with the surrounding tissue, the varying and complex inner lumen shape of the urethra (Natali et al. 2017) is simplified and defined by a parametric equation. This idealized urethra constitutes a healthy non-cylindrical-shaped lumen structure with a fibre-based nonlinear elastic behaviour. Combined with the hyperelastic balloon structure, the conducted contact simulations provide insights into the compliance-dependent shape adaption of the balloon and the resulting stress states induced to the surrounding tissue during the inflation. Due to the model build-up and parameter fitting to experimental datasets, the in-silico model is able to reveal general interdependencies in respect of low compliance ratios between the dilating balloon and targeted tissue as well as for different tissue aspect ratios.

The analysis of the conducted FEA study represents a first step towards the design of strain sensing balloon-based catheter systems for intraoperative tactile tissue diagnosis. Such systems could complement established image-based methods with intraoperative information about, e.g. the circumferential extension of plaques or quantitative compliance assessment of fibrosed vessel segments. The ultimate aim is a patient-specific treatment during established standard procedures, improving long-term outcomes. Further the present study reveals insights into basic interaction phenomena between nonlinear high-compliant balloons and non-cylindrical vessel structures. While this holds also valuable information for procedures like intra-aortic balloon occlusion procedures, it may further support the development of new types of catheter-based devices and associated interventional diagnosis or treatment options, e.g. individual stenting depending on the in vivo vessel state.

## Methods

The present FEA study was conducted within the general-purpose simulation software COMSOL-Multiphysics® version 6.0. The utilized software modules include the ‘Structural Mechanics Module’ with the additional ‘Nonlinear Structural Materials’ add-on. The computational analyses were run on a HP Z840 workstation equipped with two Intel Xeon E5-2768 v3 (Intel Corporation, Santa Clara, CA), 128 GB DDR4 RAM and a 500 GB SSD plus a 2 TB HDD disc. All 24 cores were allocated while executing the simulation. The solving process was ranging between ≈ 5 h and ≈ 74 h for a single inflation sequence dependent on the chosen balloon compliance.

To reduce run time and enable the analysis of a larger parameter range within a reasonable time frame, the 3D analysis is conducted only on a short extruded 2D section of the idealized urethra. Consequently, reported results are based on a short section in the middle of a long, regularly wrinkled vessel structure in contact with an inflating balloon catheter. An implicit solver is used for convergence purposes and to allow for dynamic analysis regarding inflation times, viscoelasticity and Mullin’s effect in future studies.

### Constitutive Modelling of the Balloon Catheter

Generally, high-pressure balloon catheters can be classified in respect of the used balloons as compliant, semi-compliant and non-compliant (Safian et al. 1995; Helou et al. 2021; Abhiram Prasad und Holmes 2013). All of these are designed as actuators for dilation of stenosed stiffened vessels and even compliant balloons are rated to pressures around 6 atm (Abhiram Prasad und Holmes 2013). In contrast, the present study investigates novel diagnostic systems with a broad range of application possibilities, where one end of the spectrum would be healthy tissue. Therefore, the term high compliant in this paper refers to compliance characteristics found in Foley catheters. These high-compliant balloons are mostly based on latex with different coatings, e.g. silicone (Lawrence und Turner 2005). Ongoing and future work in our group includes the manufacturing of balloon prototypes (Sittkus et al. 2021). The following constitutive formulation of the balloon material is based on the silicone PDMS (Sylgard® 184 Dow Corning). This enables a direct comparison of future experimental results with the current study while preserving the general characteristics of a suitable balloon material regarding Young’s modulus (kPa to single-digit MPa) and possible strain levels (> 100%).

The constitutive behaviour of hyperelastic materials is generally based on different isochoric formulations of the strain energy function (SEF) (Shahzad et al. 2015). Hereby, most of the frequently used models are phenomenological descriptions and consequently the accuracy is dependent on the used data set for parameter identification (Shahzad et al. 2015; Hopf et al. 2016). As the loading scenario for a high-compliant balloon in contact with wrinkled vessel structures may induce large and complex balloon deformations, the complexity of the model must be able to represent this behaviour considering the nonlinear nature of the balloon material. Further, the calibration set used for parameter identification of the utilized model should cover multiple strain states. For PDMS, and more specific for PDMS Sylgard® 184 in the mixing ratio 10:1 (base: curing agent), comprehensive works from Hopf et al. and Bernardi et al. established a 4-term Ogden model which covers the elastomer’s multiaxial mechanical response (Hopf et al. 2016; Bernardi et al. 2017). Hereby, the model is based on a molecular statistical reinterpretation of Ogden’s formulation by Ehret (Ehret 2015), and therefore the model can be considered to have a physical basis (in respect of the elastomers molecular chain structure) than rather being purely phenomenological (Hopf et al. 2016; Ehret 2015; Bernardi et al. 2017).

So, the constitutive formulation of the balloon structure is based on the Ogden’s model (Ogden 1972), defined by Eq. (1):

1 $$W_{ogd} = W\left( {\lambda_{1} ,\lambda_{2} ,\lambda_{3} } \right) = \mathop \sum \limits_{r = 1}^{N} \frac{{\mu_{r} }}{{\alpha_{r} }}\left( {\lambda_{1}^{{\alpha_{r} }} + \lambda_{2}^{{\alpha_{r} }} + \lambda_{3}^{{\alpha_{r} }} - 3} \right)$$

where ${W}_{ogd}$ is the strain energy density, ${\lambda }_{i}$ are the principal stretches, ${\mu }_{r}$ with the dimension of stress (Pa) are constants governing the stiffness and ${\alpha }_{r}$, dimensionless, constants considering nonlinearity in the strain-energy function, and N determines the number of terms of the strain energy function (Hopf et al. 2016; Bernardi et al. 2017). As common incompressibility is assumed and consequently ${\lambda }_{1}{\lambda }_{2}{\lambda }_{3}=1$, and Poisson’s ratio is set to $\nu =0.5$. The parameters ${\mu }_{r}$, ${\alpha }_{r}$ and the Young’s modulus $E$ (Table 1) were chosen according to the work of Bernardi et al. which is based on uniaxial, strip-biaxial and equi-biaxial testing. The utilized parameters thereby fitted best to all 3 stress–strain responses, where the least fit for PDMS Sylgard® 184 was R^2^ = 0.983 within the uniaxial loading scenario (Bernardi et al. 2017).

**Table 1:** Parameters for 4-term Ogden model according to Bernardi et al. (Bernardi et al. 2017)

Coefficient	Value
${\alpha }_{1}$	2.17
${\alpha }_{2}$	9.06
${\alpha }_{3}$	34.3
${\alpha }_{4}$	− 5.4
${\mu }_{1}$[MPa]	2.91e–01
${\mu }_{2}$[MPa]	3.40e–03
${\mu }_{3}$[MPa]	2.01e–11
${\mu }_{4}$[MPa]	− 1.15e–02
E [MPa]	1.09

### Constitutive Modelling and parameter identification for the vessel structure

#### Basic equations for idealized urethra

The common way to model the mechanical response of vessel structures utilizes hyperelastic formulations, either using isotropic models like Ogden (El Sayed et al. 2008) and Mooney-Rivlin (Prendergast et al. 2003; Auricchio et al. 2011) or the established fibre-based model known as Holzapfel-Gasser-Ogden (HGO) model, which incorporates anisotropy (Holzapfel et al. 2000). Thereby, the fibres represent dispersed families of collagen fibres where each considered vessel layer has two fibre families inclined with different angles in respect of the longitudinal axis. As the material properties of the fibres and the isotropic ground substance (including the elastin fibre response) are defined with parameters which must be determined by experiments, the model has a phenomenological component while reflecting structural characteristics of real vessels (Holzapfel et al. 2000).

The isochoric SEF ${W}_{HGO}$ of the HGO model is composed by an additive formulation of an isotropic part (${W}_{iso}$) reflecting the non-collagenous ground matrix and an anisotropic part (${W}_{aniso}$) reflecting the collagenous fibres (Holzapfel et al. 2000). Consequently, the HGO model can be written as

2 $$W_{HGO} \left( {\overline{{I_{1} }} ,\overline{{I_{4} }} ,\overline{{I_{6} }} } \right) = W_{iso} \left( {\overline{{I_{1} }} } \right) + W_{aniso} \left( {\overline{{I_{4} }} \overline{{,I_{6} }} } \right)$$

where $\overline{{I }_{1}}\left(\overline{{\varvec{C}} }\right)=tr\overline{{\varvec{C}} }$ is the first isochoric invariant of the unimodular right Cauchy-Green tensor $\overline{{\varvec{C}} }$, utilized for a Neo-Hookean formulation of the isotropic ground substance (Holzapfel et al. 2000)

3 $$W_{iso} \left( {\overline{{I_{1} }} } \right) = \frac{c}{2}\left( {\overline{{I_{1} }} - 3} \right)$$

$\overline{{I }_{4}}(\overline{{\varvec{C}} }, {{\varvec{a}}}_{01})$ and $\overline{{I }_{6}}\left(\overline{{\varvec{C}} }, {{\varvec{a}}}_{02}\right)$ are the isochoric invariants defined as the squares of the collagen fibre stretches in the direction defined by the vectors ${{\varvec{a}}}_{01}$ and ${{\varvec{a}}}_{02}$, and $c>0$ is a stress like material parameter [Pa]. With two tensors defined as ${{\varvec{A}}}_{i}:{{\varvec{a}}}_{0i}\otimes {{\varvec{a}}}_{0i}$ the invariants are consequently $\overline{{I }_{4}}\left(\overline{{\varvec{C}} },{{\varvec{a}}}_{01}\right)=\overline{{\varvec{C}} }:{{\varvec{A}}}_{1}$ and $\overline{{I }_{6}}\left(\overline{{\varvec{C}} },{{\varvec{a}}}_{02}\right)=\overline{{\varvec{C}} }:{{\varvec{A}}}_{2}$. The strain energy for the anisotropic part related to the collagen fibres is expressed with an exponential function of the form (Holzapfel et al. 2000)

4 $$W_{aniso} \left( {\overline{I}_{4} ,\overline{I}_{6} } \right) = \frac{{k_{1} }}{{2k_{2} }}\mathop \sum \limits_{i = 4,6} \left\{ {exp\left[ {k_{2} \left( {\overline{{I_{{\text{i}}} }} - 1} \right)^{2} } \right] - 1} \right\}$$

with ${k}_{1}>0$ representing a stress-like material parameter [Pa] and ${k}_{2}>0$ a dimensionless parameter.

In the newest software version of COMSOL.®, the HGO model is implemented within the ‘Nonlinear Structural Materials’ add-on. Here, the extension for a dispersion of the collagen fibres, proposed later by Gasser et al. (Gasser et al. 2006), is included. Following again the approach introduced in [46], the fibre families are complemented by a transversely isotropic free-energy function, leading to the reformulation of (4)

5 $$W_{aniso} = W\left( {\overline{I}_{4} ,\overline{I}_{6} } \right) = \frac{{k_{1} }}{{2k_{2} }}\mathop \sum \limits_{i = 4,6} \langle exp\left\{ {k_{2} \left[ {\kappa \overline{{I_{1} }} + \left( {1 - 3\kappa } \right)\overline{{I_{{\text{i}}} }} - 1} \right]^{2} } \right\} - 1 \rangle$$

with $0\le \kappa \le \frac{1}{3}$ being a dispersion parameter, where the upper limit $\kappa =\frac{1}{3}$ leads to a spherical isotropic density function (i.e. isotropic fibre dispersion) and for lower limit $\kappa =0$ Eq. (5) coincides with Eq. 4 (i.e. perfectly aligned fibres along ${{\varvec{a}}}_{01}$ and ${{\varvec{a}}}_{02}$, respectively) (Gasser et al. 2006).

It should be noted that although the polyconvexity of the constitutive equation is fulfilled for ${\overline{I} }_{4},{\overline{I} }_{6}>1$ (Holzapfel et al. 2000), the utilized split of the deviatoric and volumetric part can lead to unphysical auxetic behaviour during deformations as stated by Helfenstein et al. (Helfenstein et al. 2010). To avoid such behaviour in (Helfenstein et al. 2010) the use of the unsplit deformation gradient tensor ${\varvec{F}}$ is suggested, which was proven to be effective by Gültekin et al. (Gültekin et al. 2019). Another possibility is to apply specific constraints to the numerical scheme such as augmented Lagrangians, which results in strict incompressibility (Helfenstein et al. 2010; Gültekin et al. 2019). The HGO model implementation in COMSOL® ensures the incompressibility with a weak equation where an auxiliary pressure enforces as a Lagrange multiplier the constraint $J=1$, with $J$ being the Jacobian of the deformation gradient tensor ${\varvec{F}}:$ $J=det$ ***F*** (COMSOL AB 1). Further, a more recent publication of Fereidoonnezhad et al. (Fereidoonnezhad et al. 2020) claims to reveal the primary underlying mechanism of auxetic behaviour. Thereby, the authors state that high levels of in-plane matrix compaction as a result of increasing tension in the strain-stiffening fibres leads to unphysical out-of-plane expansion to satisfy stress equilibrium (Fereidoonnezhad et al. 2020). This also provides an explanation, why auxetic behaviour is especially high during uniaxial stretching (Gültekin et al. 2019; Helfenstein et al. 2010; Fereidoonnezhad et al. 2020) as during deformation in the 1^st^ direction the matrix compaction in the 2^nd^ direction between adjacent fibre families increases strongly (Fereidoonnezhad et al. 2020). Despite the strict incompressibility constraint, the loading scenario in the present study does not induce high levels of shear movement between adjacent fibres as only relative low levels of longitudinal stretching are applied to the vessel segments. During the subsequent inflation the relative orientation of a specific fibre family is mostly preserved and further the compensation mechanism with out-of-plane expansion, as proposed by Fereidoonnezhad et al. (Fereidoonnezhad et al. 2020), is prevented by the boundary conditions applied. Therefore, auxetic behaviour as a relevant influential factor regarding the obtained results can be excluded for the utilized loading and boundary condition within the simulations.

**Fig. 2:**
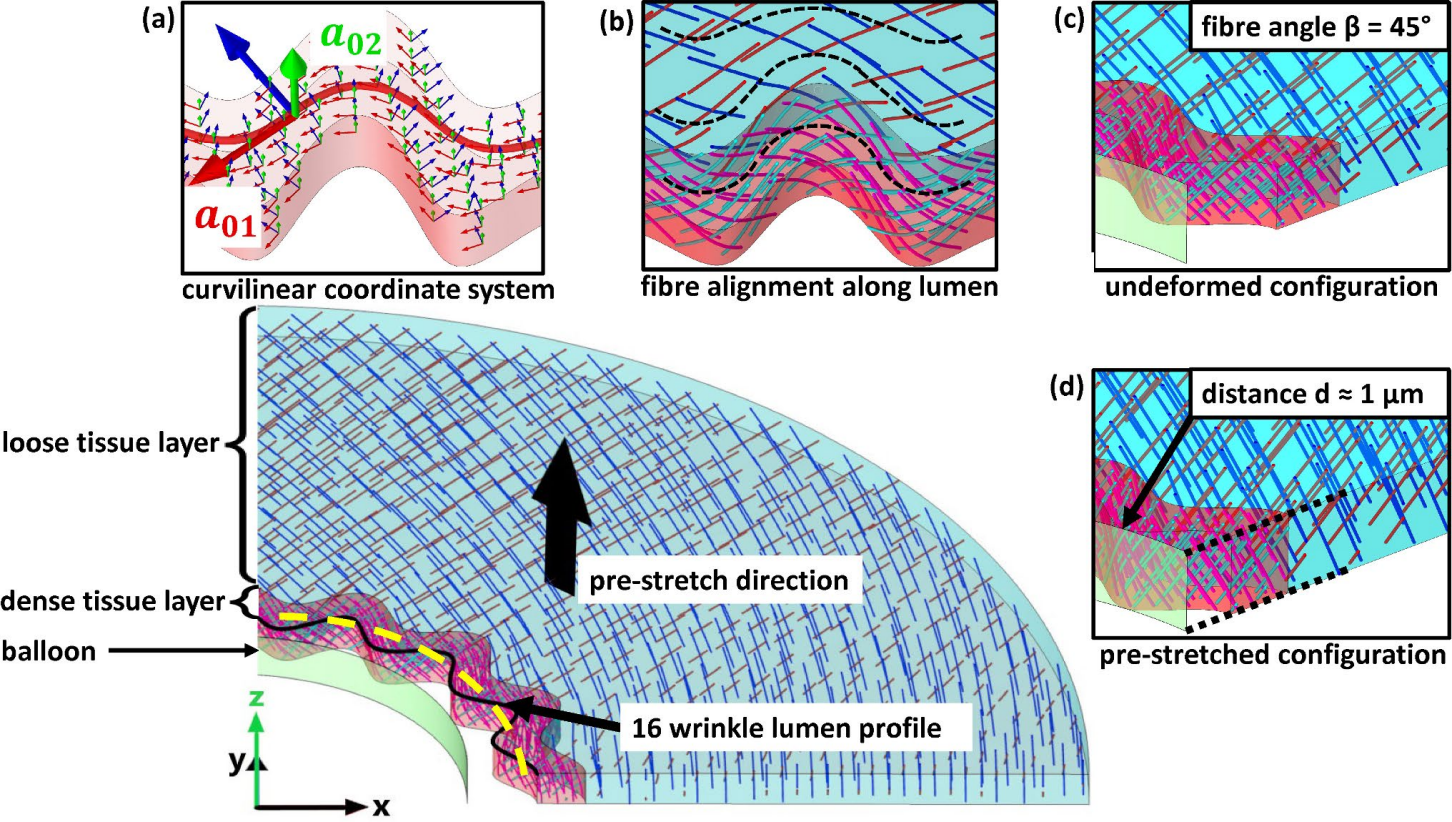
FEM model setup for a lumen profile with 16 wrinkles in the undeformed state. The mean inner radius (yellow dotted line along the lumen wrinkles), the thickness of the dense layer in radial direction and the outer diameter of the tissue are the same for each lumen profile. The fibres in the dense (red) and loose (blue) tissue layer are streamline plots representing the defined fibre orientation. **a** Visualization of the curvilinear coordinate system in the dense tissue layer, defined with the diffusion method. The directional vector ${a}_{01}$ coincides with the gradient of the computed scalar potential field (i.e. along the streamline) and ${a}_{02}$ with the z-axis of the global system (i.e. the longitudinal axis). **b** Enlarged view of the fibre alignment of the dense layer along the inner lumen. The dotted lines represent possible streamline paths of the curvilinear coordinate systems in the dense and loose layer, defined by the diffusion method. **c** Enlarged view of the undeformed state, with two fibre families in each layer with a 45° angle in respect of the longitudinal axis (z-axis) **d** Enlarged view of the pre-stretched configuration before the inflation of the balloon is started. The length of the pre-stretched vessel equals the length of the simulated balloon segment, while the initial distance between the most inner points of the lumen wrinkles and the outer surface of the balloon is individually adjusted to ≈ 1 µm for each simulated lumen profile

As the current study evaluates the balloon interaction with wrinkled non-cylindrical vessel walls, an assumption for the fibre alignment along the inner lumen is necessary. Looking at histological data provided for urethral tissue (Natali et al. 2017; Masri et al. 2018; Cunnane et al. 2021), we assume that the collagen fibres follow the inner lumen shape in parallel paths (see Fig. 2b). While Masri et al. observed an anisotropic behaviour with a stiffer response in the circumferential direction on formalin-fixated samples from human cadavers (Masri et al. 2018), Cunnane et al. found no relevant difference in the stress–strain response via uniaxial testing in circumferential and longitudinal direction (Cunnane et al. 2021). As the samples from Cunnane et al. were not preserved in formalin and were frozen immediately after excision during a male to female gender reassignment surgery, the anisotropy found within the formalin-fixated samples is attributed to the altering of the mechanical properties due to the preservation (Cunnane et al. 2021). Regarding the relative orientation of the fibres in respect of the longitudinal axis this leads to two possible assumptions: either the collagen fibres within urethral tissue are isotropic dispersed, or the fibres are aligned in an angle of 45° (angle of 90° between fibre families in ex vivo state), leading to the same response in circumferential and longitudinal direction during uniaxial testing. As in tubular vessels, collagen fibres can be considered as reinforcing structures oriented based on the physiological load scenario (Cunnane et al. 2021; Holzapfel et al. 2000), we follow the second assumption with an fibre angle $\beta =45^\circ$ in respect of the longitudinal axis, while a possible fibre dispersion is neglected, i.e. $\kappa =0$. Consequently, the collagen contribution in the urethra is modelled as a fibre containing layer with two fibre families, sub-summarizing all different layers present in the urethral wall. As the tested samples in Cunnane’s work contain surrounding looser conjunctive tissue (Cunnane et al. 2021), in which collagen fibres are present, we utilize a two-layer model where the surrounding tissue is also modelled as a fibre-reinforced material with two fibre families but a looser ground substance, similar to the FEA model of Natalie et al. (Natali et al. 2017). The layer thickness relations are extracted from data provided in Masri et al. (Masri et al. 2018) and Cunnane et al. (Cunnane et al. 2021), leading to a 250-µm-thick urethral layer beginning at the inner radius of 1.111 mm, while the looser conjunctive tissue fills the rest till the outer radius of approx. 4.352 mm is reached (relates to an averaged geometry based on Cunnane’s dataset, which are also used for parameter identification, see next section) (Cunnane et al. 2021; Masri et al. 2018).

Generally residual stresses have a high influence on the extension behaviour of pressurized vessels (Holzapfel et al. 2000). Residual stresses can be divided in circumferential residual stresses, leading to an opening angle between the cut surfaces of a longitudinal cut vessel segment, and residual stresses in the longitudinal direction, resulting to a shrinkage in length, if a vessel is extracted from its in vivo state. In Cunnane’s work only data for the residual stresses in longitudinal direction are provided (Cunnane et al. 2021). To account for residual stresses, the radius of the extracted urethra segment must be calculated based on the provided radius in the stretched in vivo state, taken from the pressure-test setup of Cunnane. With the assumption that the lumen volume confined within the cylindrical vessel remains constant during axial stretching and further isochoric assumption for the vessel this can be done by

6 $$r_{ex - vivo} = r_{in - vivo} \sqrt {\lambda_{longitudinal} }$$

where ${r}_{ex-vivo}$ represents the radius of the extracted vessel segment, ${r}_{in-vivo}$ is the in vivo radius of the physiologically pre-stretched vessel (here the data provided by Cunnane et al. (Cunnane et al. 2021) are used) and ${\lambda }_{longitudinal}$ is the ratio of the in vivo length and the extracted segment length of the same.

With the averaged values of the geometrical datasets (average ${r}_{ex-vivo}$= 4.352 mm and average ${\lambda }_{longitudinal}$ = 1.306) this leads to the aforementioned geometric parameters used to define the base dimensions of the FEA vessel geometry. Further, the assumed fibre alignment angle of 45° relates to uniaxial testing, which is also based on extracted cut-open vessel segments. Consequently, the utilized base geometry with a fibre alignment angle of 45° (Fig. 2c) resembles an extracted vessel segment of an averaged sample geometry, while possible circumferential residual stresses are neglected.

According to the assumption that the fibres follow the wrinkled lumen boundary, an appropriate coordinate system to define the fibre directions ${{\varvec{a}}}_{01}$ and ${{\varvec{a}}}_{02}$ is needed. Therefore, a curvilinear coordinate system is defined, based on the diffusion method. This ‘scalar potential method’ computes the vector field based on Laplace’s equation $-\Delta u=0$ with the vector field ${\varvec{b}}$ defined as ${\varvec{b}}=-\nabla u$. The solution is a scalar potential, and its gradient forms the first base vector of the curvilinear system (COMSOL AB 3). For needed normalization the base vectors are normalized by dividing with $\left|-\nabla u\right|$). The direction of the vector field is defined by the inlet and outlet boundary conditions, which are set on the symmetry planes of the fibre containing layers (Fig. 2a). The second base vector is defined in the longitudinal direction. In analogy to the directional definition of Holzapfel et al. (Holzapfel et al. 2000), the direction of the fibres can be defined within the curvilinear coordinate system by the directional vectors with the components

7 $${\varvec{a}}_{01} = \left[ {\begin{array}{*{20}c} {{\text{cos}}\left( \beta \right)} \\ {{\text{sin}}\left( \beta \right)} \\ 0 \\ \end{array} } \right] \, and \,\, {\varvec{a}}_{02} = \left[ {\begin{array}{*{20}c} {{\text{ cos }}\left( \beta \right)} \\ { - {\text{sin}}\left( \beta \right)} \\ 0 \\ \end{array} } \right]$$

where $\beta$ is the angle relative to the longitudinal axis, i.e. half of the angle between the collagen fibre families. Consequently, the fibre families are arranged in symmetrical spirals along the wrinkled circumferential path within the inner fibre layer and distributed over the cylindrical outer diameter in the looser outer layer (see Fig. 2b). The resulting tissue model is comparable to the model setup of Natali et al. (Natali et al. 2017), despite the utilized constitutive formulation based on the HGO fibre approach and the simplified inner lumen geometry.

#### Tissue Geometry

As the study aims for a general analysis of the aspect ratio dependent interaction with wrinkled vessel structures, the complete model geometry was defined by parametric curves (Fig. 2 + Appendix 1). In a two-dimensional Cartesian coordinate system with the spatial coordinates $x$ and $y$ the inner lumen shape is given by an equation of the general form

8 $$x_{lumen} \left( s \right) = \left[ {a + b\cos \left( {ds} \right)} \right]*{\text{cos}}\left( s \right)$$

and, respectively,

9 $$y_{lumen} \left( s \right) = \left[ {a + b\cos \left( {ds} \right)} \right]*sin\left( s \right)$$

with $a$, $b$ and $d$ being variables defining the wrinkle shape with the inner cosine function (for details see Appendix 1). To reduce the model size the parameter $s$ runs from $0$ to $\frac{1}{2}$ π. The interface curve (${x}_{interface}$) between the urethral layer and the looser surrounding tissue, can be defined in two ways. One way is a parallel curve of the form

10 $$x_{interface} \left( s \right) = x\left( s \right) + \frac{{w\dot{y}\left( s \right)}}{{\sqrt {\dot{x}\left( s \right)^{2} + \dot{y}\left( s \right)^{2} } }}$$

and, respectively,

11 $$y_{interface} \left( s \right) = y\left( s \right) - \frac{{w\dot{x}\left( s \right)}}{{\sqrt {\dot{x}\left( s \right)^{2} + \dot{y}\left( s \right)^{2} } }}$$

with $w$ being the distance parameter.

A second method to define the shifted interface path is the addition of $w$ to the parameter $a$ in Eqs. (8) and (9). This second method is not giving a real parallel curve, and therefore, the thickness of the fibre containing layer will vary when using this approach, while the first method provides a constant thickness. However, the definition according to Eqs. (10) and (11) is restricted to thicknesses which are smaller than the radius of curvature and therefore the interface curves are not touching the evolute of the lumen-defining curve. As the thickness is set to 250 µm this restricts the possible wrinkle shapes which can be simulated without having cusps. Such cusps likely lead to singularities during the solving process. To extend the possible wrinkle variance in the present study, the second method is used to define the interface of the fibre containing tissue layer, thereby accepting the thickness variance related to it. For a more detailed description of the parameter relation to the geometry of the lumen as well as the parametric definitions of the other geometric defining curves, see Appendix 1.

Here it is important to note that for certain values of wrinkle numbers the applied symmetry conditions on the x-axis and y-axis do not match with the geometric symmetry of the model. Therefore, the simulated geometries are limited to wrinkle numbers (8, 12 and 16) which result in coinciding balloon and vessel centres during inflation, and symmetry conditions match with the geometric symmetry planes. Also, the varying thickness of the fibre containing layer in combination with the utilized diffusion method to define the curvilinear coordinates leads to slight variances with regard to the parallel collagen fibre alignment (i.e. the first principal direction of ${{\varvec{a}}}_{01}$ and ${{\varvec{a}}}_{02}$, see Fig. 2) throughout the fibre layer. The resulting model geometry is exemplary shown in Fig. 1.

#### Parameter identification for the idealized urethra

As the HGO model requires the derivation of the corresponding parameters ${k}_{1}, { k}_{2}, c$ and a definition of the fibre angle $\beta$, the parameter identification is based on a cylindrical model with the provided biomechanical datasets in Cunnane et al. (Cunnane et al. 2021). The utilized approach is motivated by the works of Stalhand et al. (Stålhand et al. 2004; Stålhand und Klarbring 2005) and Heusinkveld et al. (Heusinkveld et al. 2018). The complete identification procedure was conducted within the commercial software MATLAB® (v2021a). The parameter identification procedure within this study aims for a tissue response which reflects the response of a healthy urethra. As the database is limited and a cylindrical geometry simplification is necessary for an effective problem formulation, we have chosen an averaging method based on 6 samples. Details about the procedure can be found in Appendix 2, and the full data calculated based on Cunnane’s dataset as well as the corresponding MATLAB® code are provided in the supplementary data (**Online Resource S1, Online Resource S2** and **Online Resource S3**).

The obtained parameters are 3.5735 for the matrix stiffness parameter ${c}_{d}$ of the dense layer and 1.7180e-02 for the loose layer ${c}_{l}$, 1.5966 for the ${k}_{1d}$ parameter of the dense and 7.6762e-03 for ${k}_{1l}$ of the loose layer, and 0.1711 for the ${k}_{2}$ parameter which is set equal for both layers (see also Table 2, Appendix 2).

### Solver setup, contact definition, boundary conditions and meshing

Even though the conducted analysis is quasi-static and effects like viscoelasticity are not included, the solving process is implemented as time-dependent study as this provides easy options to fine tune solver settings for a better convergence. Further it allows to increase the pressure load on the inner balloon surface till either a loss of numerical convergence leads to an abortion of the simulation or a final time/max. pressure level is reached. To enhance stability and avoid higher levels of interpolation, the solver stepping was set to “strict”, which limits the maximum step size while allowing intermediate steps if necessary (COMSOL AB 3). To restrain possible solver time steps which may lead to convergence issues the time stepping was set to 0.001 s. The pressure load was ramped according to a linear function (R(t): ℝ → ℝ_0_^+^) of the general form R(t) = t, defined in the time (t) domain (viz. 1 s equals 1 kPa pressure). The pressure was applied on the lumen side of the balloon. Beforehand, the undeformed vessel segment is pre-stretched with a prescribed displacement (also with a ramped function in the time domain) to the averaged value 1.306.

**Fig. 3:**
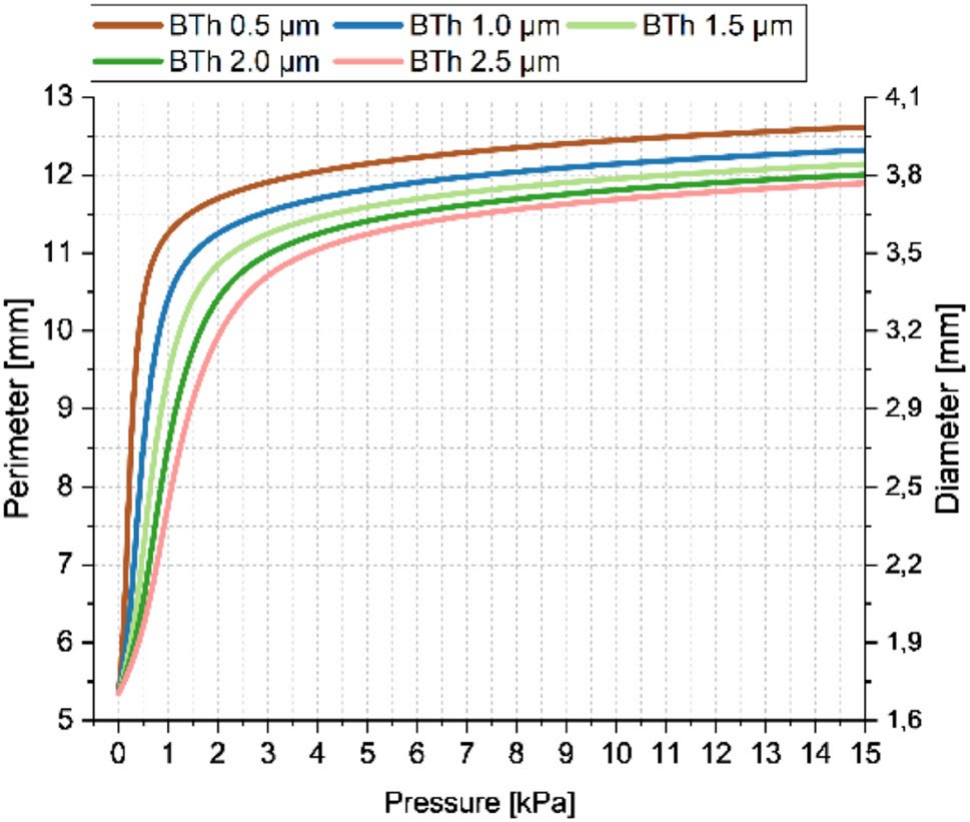
Simulated inflation up to 15 kPa of circular balloon structures with 5 varying PDMS thickness values between 0.5 and 2.5 µm in 0.5 µm steps

The high balloon compliance implies that the pressure level utilized for the inflation of the balloon is around one to two orders of magnitude lower than for standard compliant balloons. The analysed pressure range up to 15 kPa is thereby motivated by the simulated balloon inflation behaviour of the PDMS balloons (see Fig. 3). Here, the typical expansion behaviour of hyperelastic elastomer materials can be observed, where at higher pressures the stiffening can be related to non-coiled elastomer fibres. For the standard PDMS Sylgard® 184 mixing ratio of 10:1 (base: curing-agent), the maximum tensile strain is around 140% (Jang et al. 2016), which is reached at around 15 kPa for the utilized 4-term Ogden model and parameters of Bernardi et al. (Bernardi et al. 2017). The thickness values were estimated iteratively during preliminary simulations, while the lower bound of 0.5 µm is chosen due to decreasing convergence levels and experimental boundaries during classical manufacturing of PDMS membranes (as spin coating without diluting the PDMS material with hexane is limited to around 1 µm) (Firpo et al. 2015). The starting distance between the deflated balloon and the pre-stretched inner vessel wall was manually adapted for each setup to ≈ 1 µm. This is important to ensure comparability of the results, as for a contact after substantial balloon inflation the emerging circumferential tensile stresses within the balloon wall lead to different “initial” stress states compared to lower inflation values at contact.

The contact pair is defined on the outer side of the balloon and the lumen side of the tissue. The contact feature within COMSOL® has two algorithms available. For the present study the ‘Augmented Lagrangian method’ is used, as it enforces the non-penetration condition by using weak constraints and Lagrange multipliers. This leads to more accurate results compared to the ‘Penalty method’ where overlapping occurs (COMSOL AB 2). To reduce the computational time and based on the assumption that the lumen surfaces are fully hydrated and shear load transfer has a negligible impact for the chosen geometries and the corresponding deformation behaviour in the analysed interaction range, the contact is simulated without friction. As just a quarter of the vessel segment and balloon is simulated, symmetry conditions apply to all boundaries of the balloon as well as the tissue segment except the inner (lumen side) and outer surface of the tissue and balloon, respectively.

**Fig. 4:**
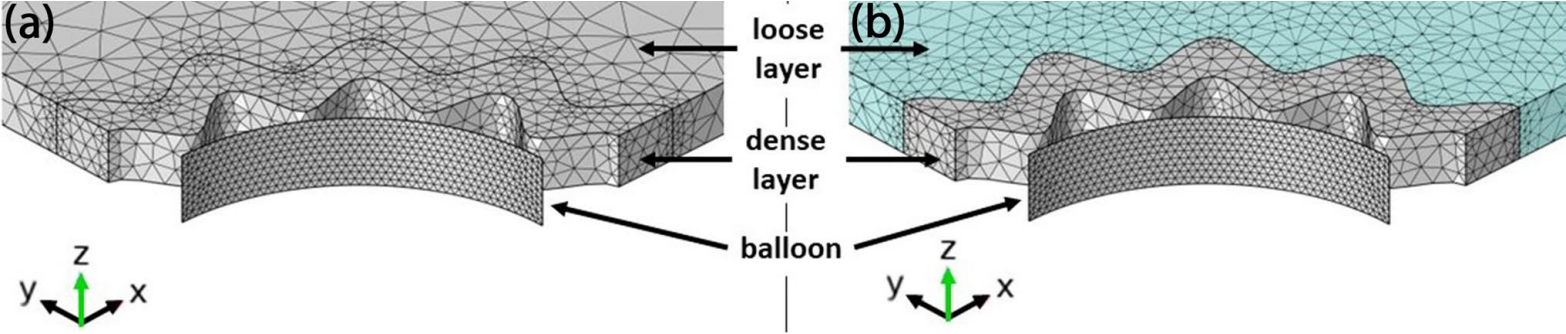
Exemplary mesh section of the starting mesh and the final re-meshed solution via adaptive mesh refinement. **a** Starting mesh for a balloon thickness of 1 µm and a tissue geometry with 16 folds. The balloon has 922 triangular mesh elements extruded in radial direction, the denser tissue layer has 4899 and the looser outer layer has 4383 tetrahedral mesh elements. **b** Final re-meshed solution after 4 iterations. While the element count in the balloon and the denser tissue layer remains the same, the looser outer layer element count increased to 23536 elements (cyan coloured region)

For a two-body contact problem with similar stiffness properties and large deformations an incremental mesh refinement is reasonable, as with larger deformations the mesh element quality can quickly become poor, ultimately leading to a non-converging simulation. Details about the implementation in COMSOL® can be found in Appendix 3. The starting mesh consists of two different element types, where the balloon is meshed with a free triangular surface mesh which is extruded along the radial direction, while the tissue is meshed with free tetrahedral elements where the denser tissue layer is meshed equal or finer than the looser outer layer (see Fig. 4). For all elements the default cubic shape function (second-order (quadratic) discretization) is used. The adaptive mesh refinement was implemented for each simulated geometry up to a maximum of 8 iterations (see Table 3 in Appendix 3). Details about the utilized meshes as well as a mesh refinement study for a representative model (Figs. 13 and 14 in Appendix 3) can be found in Appendix 3.

### Analysis methods and parameters

As for different balloon thicknesses and tissue geometries varying shape adaptions of the balloon can be observed, two unitless control parameters are introduced for comparison:

- The first one describes the relative area of the outer balloon surface in contact with the tissue at different inflation pressure levels. In the following this is referred to a so-called Conformal Contact Factor (CCF) which depends on the pressure ${P}_{input}$ and the balloon thickness (BTh). Thereby, the relative contact area is given as relative value to the current total outer balloon surface at the corresponding pressure level, which leads to
  12 $$CCF\left( {P_{input} \left( i \right),{\text{ BTh}}} \right) =  {1 - \frac{{({\text{BA}}\left( {\text{i}} \right) - {\text{CA}}\left( {\text{i}}) \right)}}{BA\left( i \right)}} $$

where $BA(i)$ is the respective total outer balloon surface and $CA(i)$ is the respective outer balloon surface in contact with the surrounding tissue at the corresponding pressure level. Consequently, for a perfect conformal adaption of a balloon to the surrounding tissue shape, $CCF$ becomes 1, while after contact $CCF$ is always $>0$.

- The second control parameter describes the relative deviation of the balloon from an idealized cylindrical shape during an inflation cycle and is also depended on $BTh$ and the inflation pressure ${P}_{input}$. In the following this is referred to as ${P}_{input}$ and $BTh$-dependent Balloon Adaption Factor (BAF). Thereby, the relative deviation is defined as the ratio between the perimeter of an idealized cylindrical shape based on the most inner contact points between balloon and tissue, and the perimeter of the adapting balloon shape for a 2-dimensional axial cut and is given by
  13 $$BAF\left( {P_{input} \left( i \right), BTh} \right) = \frac{{{\text{BPer}}_{adapt} \left( {\text{i}} \right)}}{{{\text{BPer}}_{ideal} \left( {\text{i}} \right)}}$$
  where ${\mathrm{BPer}}_{adapt}\left(\mathrm{i}\right)$ is the current outer balloon perimeter and ${\mathrm{BPer}}_{ideal}\left(\mathrm{i}\right)$ is the idealized cylinder perimeter. Consequently, a $BAF$ of 1 means that the inflating balloon retains its cylindrical shape, while $BAF$ > 1 means a deviating balloon shape.

For details about BAF determination, see Appendix 4.

## Results

**Fig. 5:**
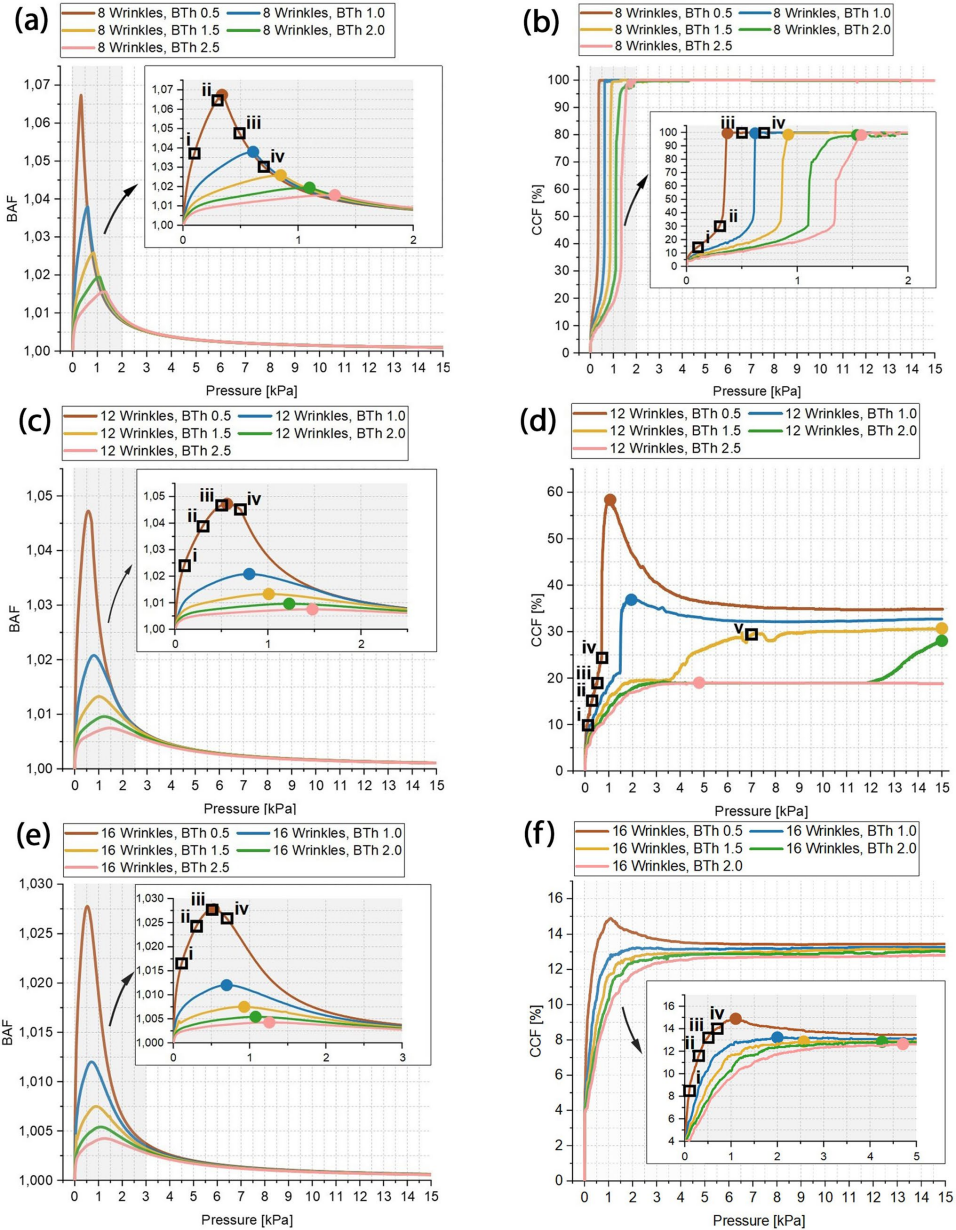
Balloon thickness (BTh) dependency of BAF and CCF in contact with the 8 **a**, **b**, 12 **c**, **d** and 16 **e**, **f** wrinkle tissue model. Insets in **a**, **b**, **c**, **e** and **f** show details within low-pressure range. Coloured bullet-point symbols mark max. values of each curve. The boxes i-iv are measurement values used in Fig. 6. The mesh-related inaccuracies in **d** are addressed in Appendix 3

**Fig. 6:**
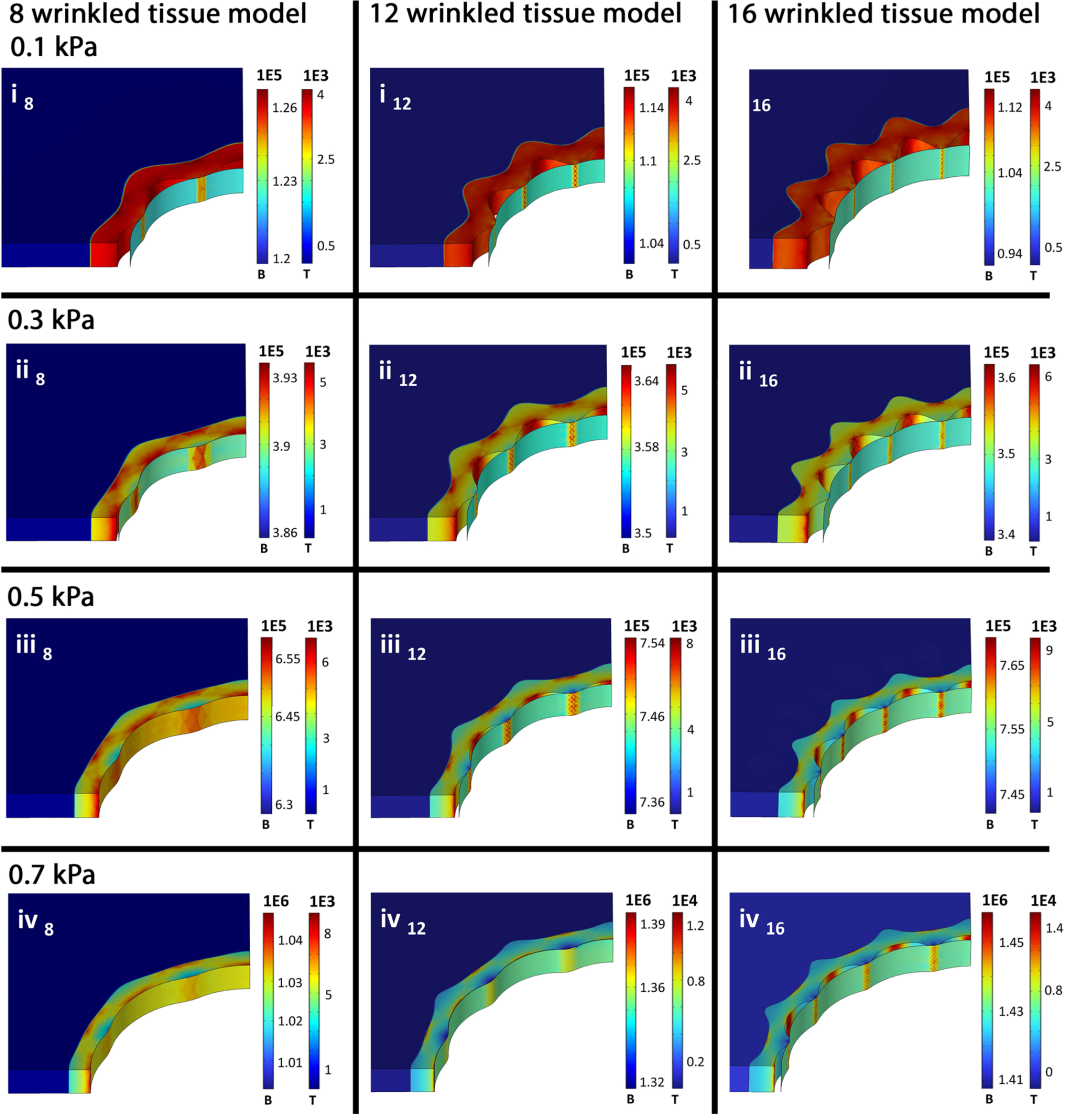
Low-pressure balloon-inflation plots of the first principle stress inside the balloon and tissue for a balloon thickness of 0.5 µm in contact with 8, 12 and 16 wrinkle tissue. The plots shown are generated at pressure values indicated by the box symbols within Fig. 5**a**–**f**. Colour legend: B: balloon, T: tissue. All units of the colour scales are in $\left[N/{m}^{2}\right]$

**Fig. 7:**
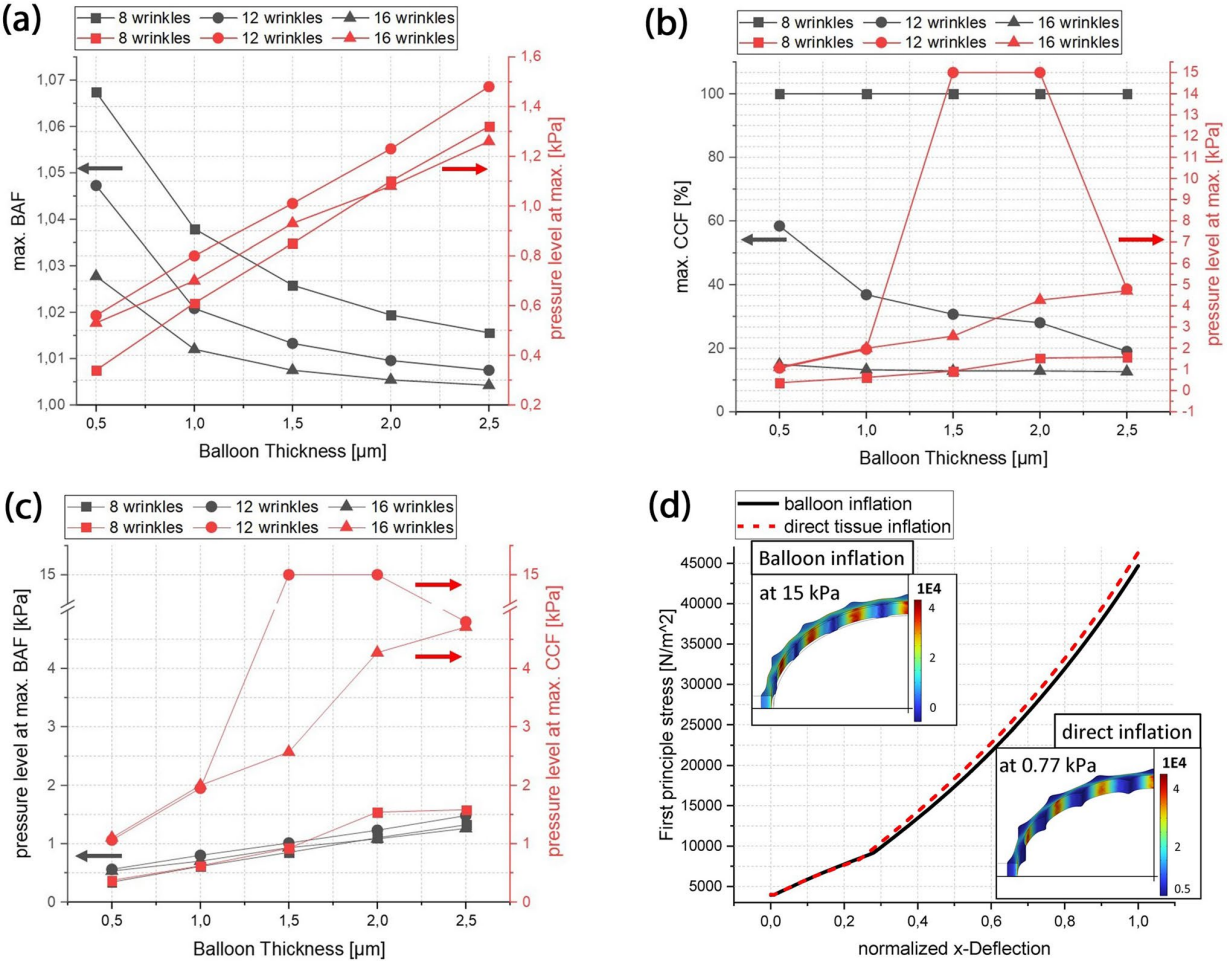
Extracted parameters from simulations for 8–16 wrinkles as function of balloon thickness and stress comparison between direct tissue inflation and balloon-induced tissue deflection. Maximum BAF **a** and CCF **b** values for different tissue models in dependence of the balloon thickness and the corresponding inflation pressure at the observed maximum. The shown data points correlate with the bullet-point symbols in Fig. 5. **c** Comparison of inflation pressures at max. BAF and max. CCF values in dependence of the balloon thickness. The y-axes are cropped for better visibility. **d** First principle stresses inside the 16 wrinkle dense tissue layer in dependence of the normalized deflection in-x-axis direction. The insets show the corresponding max. deflection states of the dense tissue layer. The values of the colour legends are in $\left[N/{m}^{2}\right]$

In total 17 simulations (5 balloons with 3 different tissue models + 2 simulations with varying tissue parameters) were analysed. In Fig. 5 BAF and CCF in respect of the inflation pressures up to 15 kPa are presented for each tissue model with averaged tissue parameters and balloon thickness. The plots are generated based on 10 Pa steps. The marked boxes refer to Fig. 6 in which exemplarily the corresponding balloon deformations are shown. Here, each column corresponds to one tissue model and the rows correspond to 100, 300, 500 and 700 Pa pressure. The colour legends correspond to the first principle stress distribution inside the balloon and tissue structure. The legends were scaled for better visibility. Figure 7a, b show the extracted maximum BAF and CCF values of each simulation in dependence of the balloon thickness as well as the corresponding pressure level at the maximum. Figure 7c summarizes the corresponding pressure values at the maximum for BAF and CCF. Figure 7d shows the first principle stress inside the 16 wrinkle dense tissue layer with averaged tissue parameters as function of the normalized deflection in x-axis direction, where a direct tissue pressurization is compared to a tissue deflection induced by a balloon inflation (BTh 0.5). For this, the deflection of an edge point along the symmetry plane was taken for comparison.

**Fig. 8:**
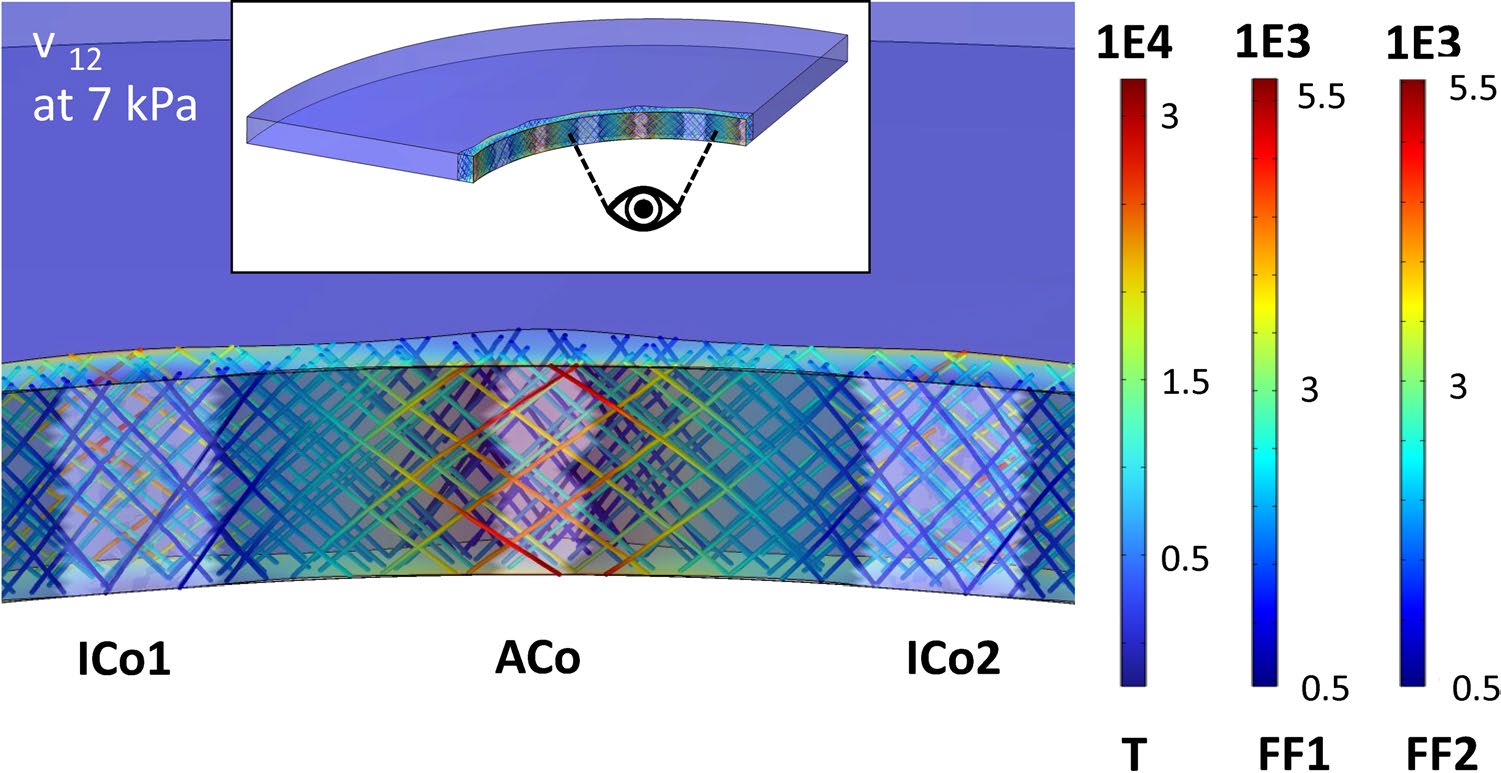
Second transition phase from the initial contact to the final contact state observed for a balloon thickness of 1.5 µm and the 12 wrinkled tissue model with averaged tissue parameters (point v in Fig. 5d). The colour legends refer to the first principle stress inside the tissue (T) and the fibre stresses of the two fibre families (FF1 and FF2), represented by thin lines within the plot. All units are in $\left[N/{m}^{2}\right]$. The white shaded areas are evaluated based on an in-contact variable within the FEA software and corresponds to the contact areas. Inset shows view direction of enlarged area in respect of full model

**Fig. 9:**
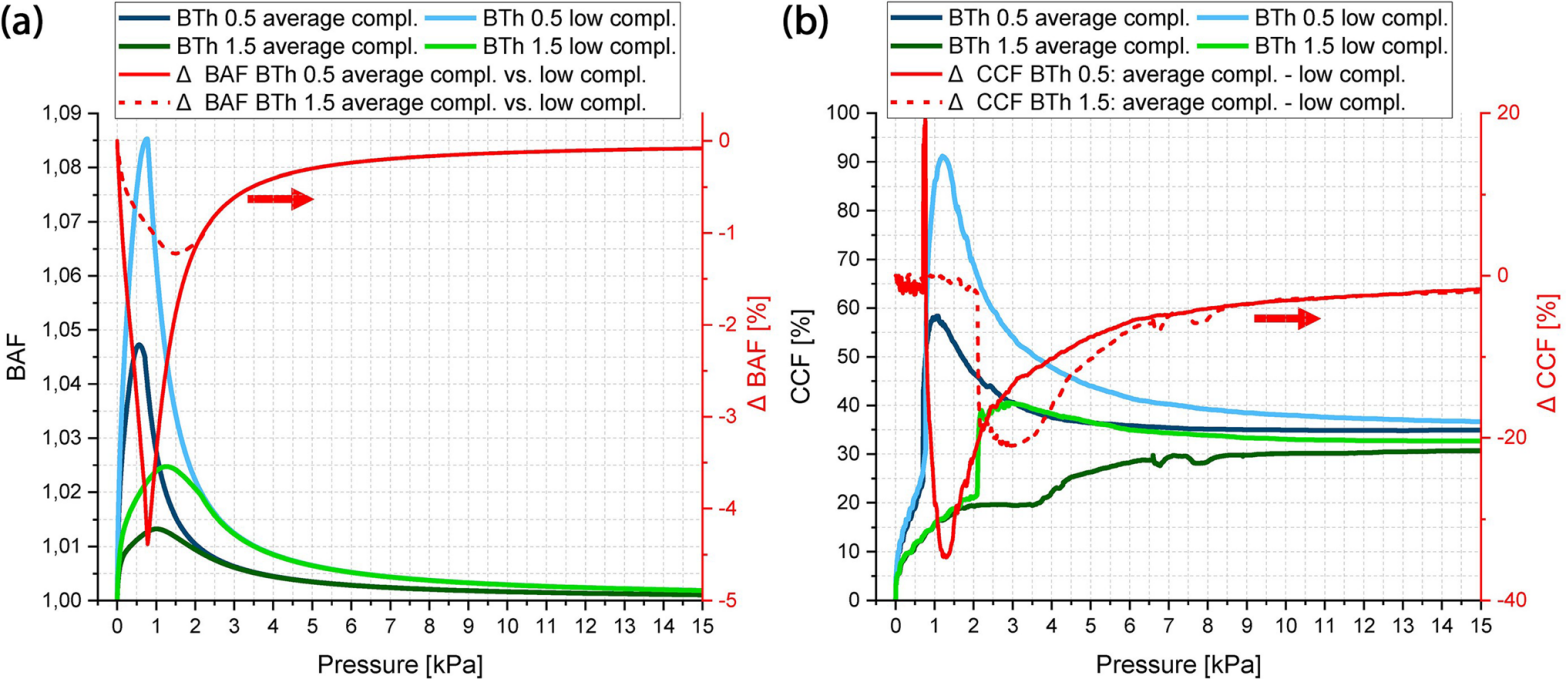
Tissue compliance dependency of BAF **a** and CCF **b** for the contact of a high-compliant (BTh 0.5 µm) and intermediate-compliant (BTh 1.5 µm) balloon with the 12 wrinkle tissue model. Thereby, the average compliance refers to the utilized tissue parameters for all other presented results (Appendix 2, Table 2 last row), while the low-compliant tissue parameters are corresponding to the stiffest tissue sample 1 of the utilized Cunnane dataset (first row parameters in Table 2 Appendix 2). On the right axis the relative difference of the BAF parameter in respect of the averaged parameter set for **a**, and the difference by subtraction between the averaged and low-compliant CCF factor are shown. The mesh-related inaccuracies in **b** are addressed in Appendix 2

Figure8 shows a deflection state, where the maximum balloon deflection area (i.e. the midpoint between the initial contact sites (ICo)) comes in contact with the tissue wall and an additional contact area (ACo) evolves. The plotted state corresponds with the box symbol area ‘v’ in Fig. 5 d. In Fig. 9 the influence of a varying tissue compliance on BAF and CCF is shown for a high and intermediate balloon compliance in contact with the 12 wrinkle tissue model. On the right axis the relative differences are shown, either with respect to the BAF value for the averaged tissue parameters in (a) or the difference resulting from subtraction for the corresponding CCF values in (b). The meshing of the models remained thereby unchanged.

## Discussion

As shown in Fig. 5, the control parameters BAF and CCF are useful descriptors for the interaction behaviour between high-compliant balloons and the surrounding winkled tissue. Since the balloon wall thickness is directly related to the compliance of the balloon, the introduction of balloon compliance values is waived within the present work, as within any method of extracting such values certain boundary conditions (e.g. the specific values taken for calculation) would impede a comparison.

The present study analyses a relatively large dataset, while the computational time was limited with relatively coarse tissue meshing, leading to inaccuracies within the obtained results. As the study aims at general interdependencies which can be used to obtain design guidelines for sensing high-compliant balloon catheters, the following discussion relates mainly to semiquantitative interdependencies and trend analysis. Shown in Appendix 3, these relations hold also true for the coarser meshes. The 3 analysed tissue geometries correspond to 3 different aspect ratios regarding the ratio between width and depth of the tissue wrinkle. In the following these tissue models are representing low aspect ratio (8 wrinkles), intermediate aspect ratio (12 wrinkles) and high aspect ratio (16 wrinkles).

### General aspects, approaches to obtain lumped models and tissue stress distribution

**Fig. 10:**
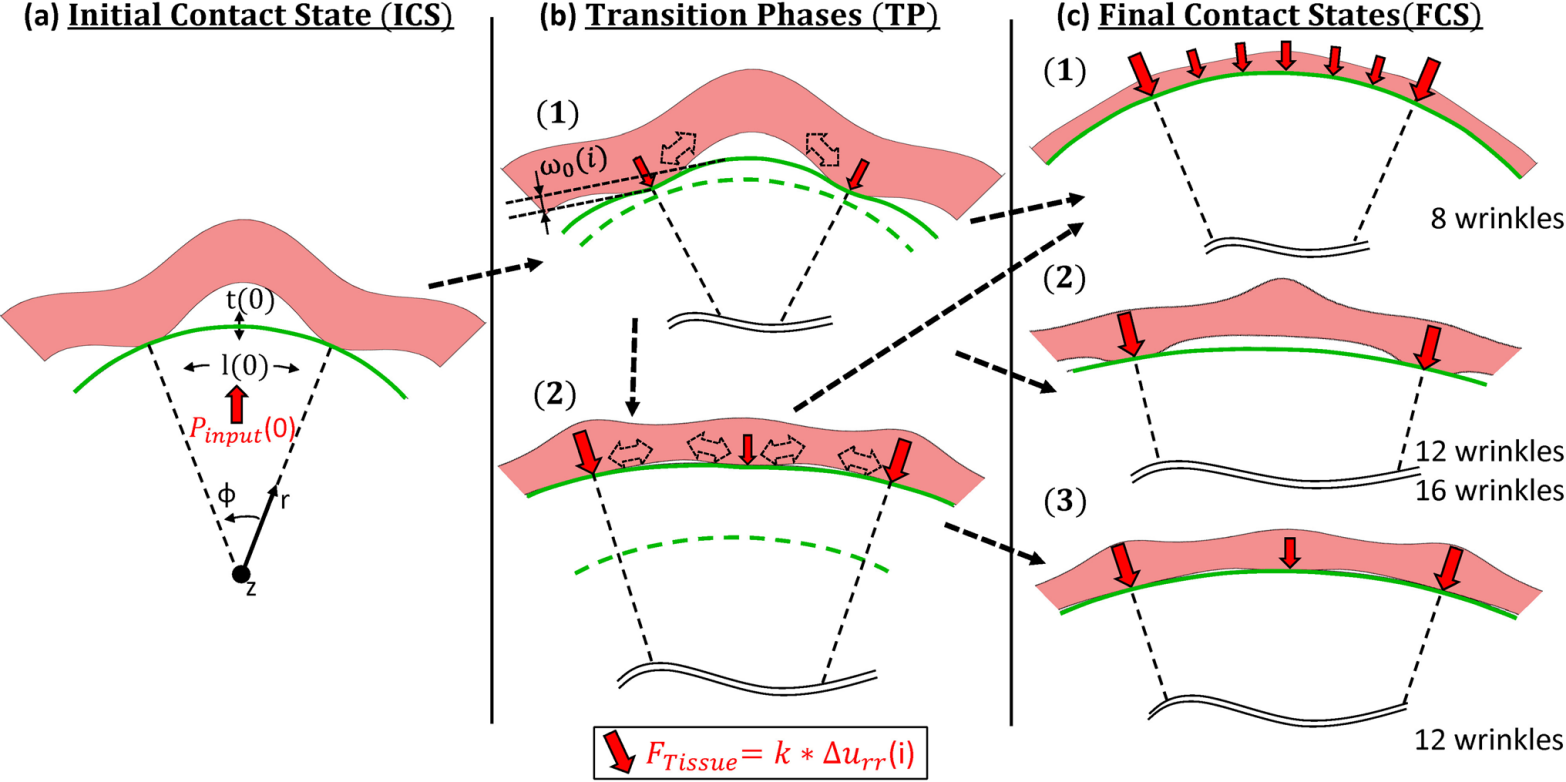
Schematic of interaction states and transition phases observed in the present study. The problem is scaled down to an one-wrinkle balloon interaction. **a** ICS as starting point for the treatment as thin plate or membrane deflection problem in a cylindrical coordinate system, with adapted parameters, namely current (starting) membrane thickness $t(0)$, current membrane length $l(0)$ and starting pressure ${P}_{input}(0)$. **b** TP from initial contact to final contact states, with the current maximum deflection ${\omega }_{0}$(i) in dependence of the relative input pressure ${P}_{input}\left(i\right)- {P}_{input}(0)$. The 2-directional dotted arrows account for enlarging and due to balloon stiffening decreasing contact areas. The dashed line represents the plate/membrane shape at the initial contact. The given tissue force ${F}_{Tissue}$ as function of the isotropic matrix stiffness $k$ and the relative radial displacement $\Delta {u}_{rr}\left(i\right)$ only accounts for an isotropic tissue treatment. **c** Final contact states for the different tissue geometries, while in the current study the 3^rd^ state was just observable after TP 2. Note that while the initial contact points coincide with the highest radial tissue force, the other forces along the contact sites differ based on the TP from ICS to FCS and also do not account for stiffening due to fibre stretch

Looking at the CCF values in comparison with the inflation plots, it can be distinguished between different interaction sequences: Between the initial state and the ‘final’ contact states 2 different transition paths exist. The ‘Final’ Contact States (FCS) at the maximum balloon inflation are mainly dependent on the tissue shape. Scaling it down to one single wrinkle interaction, the inflation after the Initial Contact State (ICS) can be correlated to a rectangular thin plate/membrane deflection problem, where the membrane edge segments are non-rigid and expand radially outwards (Fig. 10b 1). Consequently, this phase is present for all balloon tissue combinations. Dependent on the aspect ratio of the tissue, the transition paths (TP) differ, where in one case the contact is gradually enlarging from the initial contact points. The 2^nd^ transition occurs at a certain deformation state of the tissue, and an additional contact region evolves at the maximum deflection area of the ‘thin plate/membrane’ (see Fig. 8 and Fig. 10b). This behaviour is enforced by the fibre stiffening during the deformation of the tissue, leading to strong stiffening at high tensile stress/strain areas. This evolving additional contact area at the middle section between the two initial contact sites is most prominent for balloon inflations in contact with an intermediate tissue aspect ratio (Fig. 5d) and Fig. 8), where higher balloon thicknesses lead to a contact at the middle section at higher pressure levels.

Despite the enlarging contact area during balloon inflation, for high complaint balloons in contact with intermediate or high aspect ratio tissue models, also a decreasing CCF is observable (Fig. 5d, f). This is directly related to the stiffening of the balloon wall with increasing inflation due to the evolving circumferential tensile stresses. Thereby, the maximum BAF is reached in the high-compliant region of the balloon (up to around 2 kPa for the current balloon material and thicknesses, Fig. 5a, c and e). At higher pressure levels the balloon returns back to a more cylindrical shape. This behaviour is observable for every balloon tissue combination and can be regarded as a general behaviour of balloon catheters. In interventional balloon-angioplasty procedures this can be related to the well-known dog-boning behaviour, whereby the dog-boning effect is decreasing with increasing inflation pressure. While in such procedures the deformation of the balloon is regarded as unwanted effect, for sensing balloon catheters motivating this study, the effect of balloon shape deformation is necessary, especially if the diagnosis also aims for the detection of the lumen shape (e.g. to analyse the plaque spread in circumferential direction).

The FCS differs based on the TP and a full contact is just obtained for tissue geometries with lower aspect ratios. Aiming at a lumen shape detection by analysing the balloon deformation, this imposes certain limitations. To find suitable lumped reverse models, for the current model setup it is reasonable to relate the interaction to a thin plate/membrane deflection problem (Fig. 10). However, especially for small plaque balloon interactions expected within pathological cylindrical blood vessels, it may be more reasonable to relate the deformation to an indentation problem with a spring loaded indenter, while the spring characteristics are defined by the surrounding vessel and the plaque is taken as a solid, incompressible indenter.

As shown in Fig. 10, the maximum deflection area allows to define the middle point of a tissue wrinkle. Looking at the TP, for wrinkles with low aspect ratios the maximum deflection coincides with the (deformed) wrinkle depth (Fig. 6 (${iii}_{8}$) and Fig. 7c) and the deformed thin plate/membrane shape resembles the deformed tissue shape. Situations, where this full contact is established at low-pressure values, can be regarded as ideal case for a tissue shape determining balloon catheter. Dependent on the compliance ratio between the balloon and the tissue, a non-uniform membrane deflection path must be taken into account for low aspect ratios as the evolving tissue contact is leading to a deviating membrane shape (Fig. 10b1). Further, as soon as a TP with an evolving additional contact area at the middle section occurs (Fig. 10b2), the membrane deflection analogy is no longer applicable.

For certain tissue balloon combinations the neglection of the evolving tissue contact, associated with the treatment as thin plate/membrane deflection problem, may lead to pronounced deviations from the real tissue shape. This emphasises the need for reverse models which are able to estimate the deformation path and consequently are also able to map the obtained deforming balloon shape to the undeformed initial tissue state. Despite this, the maximum BAF values in dependence on the BTh (Fig. 7a) show that in agreement with the thin plate theory, a cubic relation for increasing BTh values can be assumed during the TP (Fig. 10b 1). Associated with this, He et al. (He et al. 2022) recently revisited the classical treatment of the Föppl-von Kármán equations used to describe thin plate/membrane deflections and addressed the related basics of moderately large deflections and small rotations. To overcome the issues associated with the small-rotation-angle assumption they reformulate the classical equations without this assumption and show that for cylindrical bending the problem can be associated with an one-dimensional beam (thin plate) or cable (membrane) problem leading to analytical solutions with the help of the perturbation method (He et al. 2022). As the ICS in the current study is relatable to a cylindrical bending problem with large deflections and large rotations, the obtained relations from He et al. (He et al. 2022) may serve as suitable starting point for lumped models and reverse identification procedures.

Despite the shape analysis of vessel structures, the proposed tactile sensing balloon catheters must also be able to determine the stiffness of the surrounding tissue. Thereby, it is reasonable to take the compression of the tissue at the initial contact points during the TP. Based on the assumption that the collagen fibres do not contribute to the compression and in initial phases the compliance of the compressed tissue is solely influenced by the isotropic matrix, the obtained stiffness values in the low-pressure regimes can be related to the Young’s modulus of the isotropic matrix and at higher inflation levels to the nonlinear collagen stiffening behaviour. Looking at obtainable differences in respect of the utilized BAF and CCF value, the comparison with a tissue segment with lower compliance (i.e. sample 1 Table 2) reveals a pronounced difference in respect of the interaction sequence. As expectable, a stiffer tissue thereby leads to higher BAF values, while the magnitude decreases with decreasing balloon compliance (i.e. increasing BTh, Fig. 9a). Further the maximum obtainable relative contact area increases and the CCF curve progression changes. Thereby the pressure level at the maximum CCF value decreases for an intermediate balloon compliance and the CCF curve progression resembles the curve shape of a higher compliant balloon (Fig. 9b, for comparison BTh 1.5 to BTh 1.0 in Fig. 5d).

The observable stress distribution inside the dense tissue layer (opposing to the tissue geometry, see Fig. 6, Fig. 7d and Fig. 8) can also be seen during direct inflation of the tissue and is consequently associated with the non-circular tissue geometry itself. Taking a closer look to the presented stress states in the FEA study of Natali et al. (Natali et al. 2017) on a real urethral geometry, similar stress fields originating from the negative parts of the tissue wrinkles (with respect to the lumen centre) are observable at low pressures. The origin of such stress distributions can be assigned to the radial expansion of wrinkled non-circular tissue, leading to increased tensile stress levels in such wrinkle valleys. Further, stress sites evolve also at the lumen orientated wrinkle parts on the interface to the looser surrounding tissue. The collagen fibres thereby lead to an additional exponential stiffening due to the evolving tensile stresses (compare Fig. 8) and a more confined stress site formation (compared to isotropic models) can be assumed. With further research on the 3-dimensional real collagen fibre alignments, detailed FEA analysis of possible rupture sites during PTA procedures or urethral dilatation procedures is also possible.

Looking at the obtained stress levels, even in the high-compliant balloon region (inflation at low pressures), the stress states inside the tissue remain around 2 orders of magnitude lower than within the balloon wall (Fig. 6). The tissue stress is mainly compensated inside the denser inner layer and reduces quickly within the surrounding porous soft layer while, as mentioned prior, a high stress path is formed at higher pressures in an opposing manner compared to the tissue geometry (visible in Fig. 6 at 0.5 and 0.7 kPa and Fig. 8). Critical stress levels leading to possible tissue damage consequently form within the negative parts of such tissue wrinkles and on the interface to the surrounding loose layer at the initial contact sites between the balloon and tissue. However, as the stress levels for comparable deflection levels are similar for a direct tissue inflation and the corresponding deflection induced by a balloon (Fig. 7d), the use of sensing balloon catheters can be regarded as safe at low-pressure/high-compliance regimes.

Even the current study is based on idealized tissue geometries, some general semiquantitative relations can be summarized from the presented results. The following relations also apply for more complex lumen shapes, e.g. present in a real urethral lumen.

1. The local balloon tissue interaction is determined by the balloon compliance and the tissue aspect ratio, where a full contact between balloon and tissue is only obtainable for low tissue aspect ratios.
2. A lower tissue compliance leads to higher balloon deformations and a higher maximum contact area. Consequently, the relative compliance ratio between the balloon and tissue determines the interaction sequence.
3. The balloon tissue contact surface evolves in two different ways, where for intermediate tissue aspect ratios an additional contact side evolves at the maximum balloon deflection area which is enforced by collagen fibre stretching. For high tissue aspect ratios the contact surface evolves gradually from the initial contact sites due to tissue compression and balloon deformation, where for lower balloon thicknesses (viz. higher balloon compliance) generally higher maximum contact areas are obtainable.
4. The balloon stiffening due to the evolving tensile stresses inside the balloon wall can lead to a decreasing contact area with the surrounding tissue. This occurs for intermediate and high aspect ratios within the tissue.
5. The maximum balloon deformation is obtained at low-pressure regimes where the tissue shape is imprinted to the balloon, while at higher pressure levels the balloon goes back to a more cylindrical shape. This is the case for all tissue aspect ratios and is comparable to the well-known dog-boning effect in balloon-angioplasty procedures. Thereby the interaction between two contact sites can be described as thin plate/membrane deflection problem, where at higher pressure levels increasing tensile stresses inside the balloon wall lead to a decreasing maximum deflection.
6. As it is reasonable to operate such sensing balloons within the low-pressure/high-compliance regime, the use is likely not harming the surrounding tissue as long as the deflection is in the range or below the physiologically occurring deflections.

### Design guidelines for sensing balloon catheters

Literature related to sensing multifunctional balloon catheters discusses all kind of complex sensors inside such devices (e.g. (Han et al. 2020)). In this work the motivation originates from high-compliant strain sensing balloon catheters which are able to tactilely map the vessel surface with respect to shape and stiffness. This is of special interest for common dilatation procedures as dilatation pressure and e.g. the necessity of subsequent stenting could be evaluated specifically for the individual patient.

**Fig. 11:**
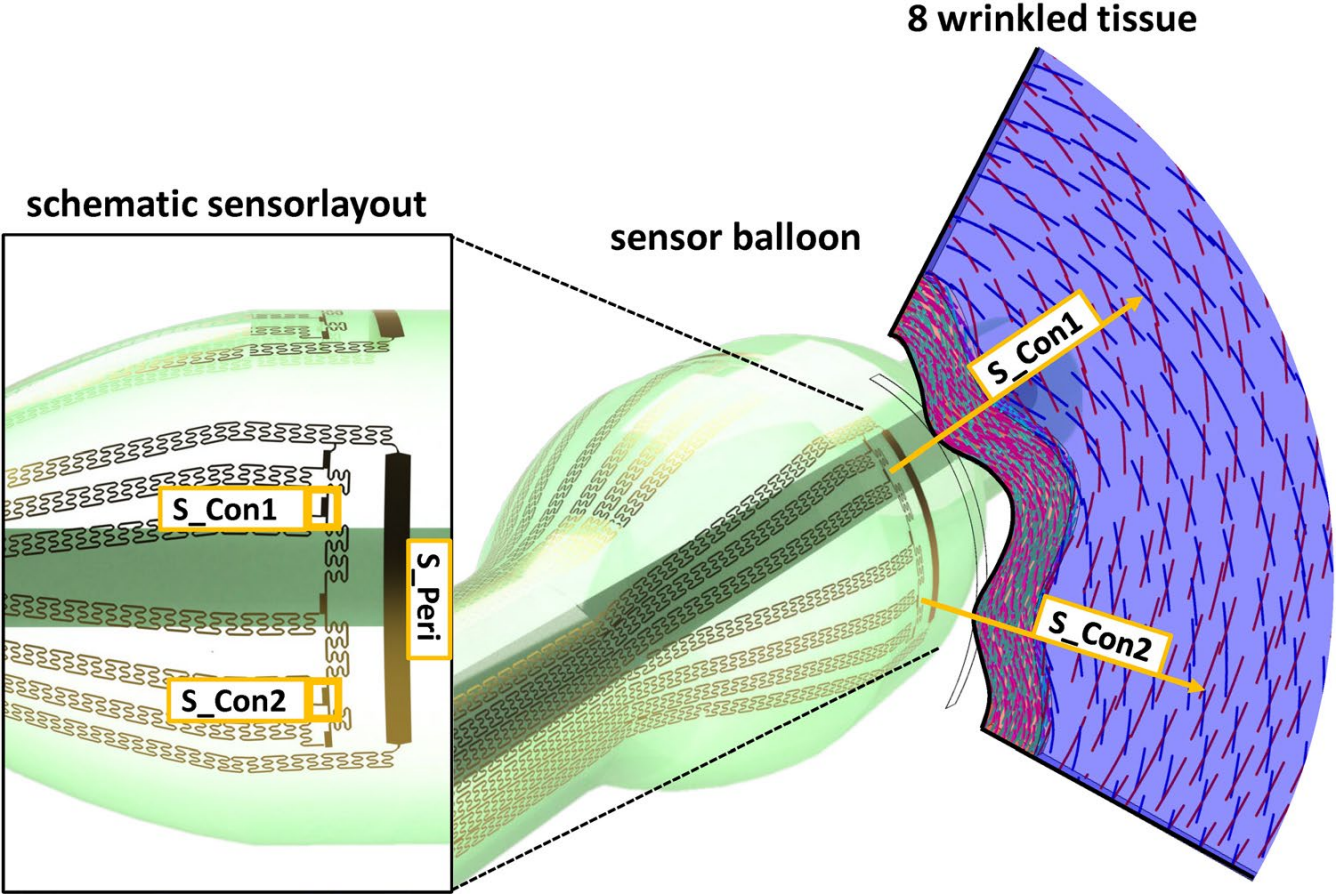
Exemplary balloon–tissue interaction with a simplified balloon sensor layout in contact with a 8-wrinkeld tissue geometry. Thereby, the contact strain sensors S_Con1 & S_Con2 provide the initial contact points and the corresponding evolution, while the perimeter strain sensor provides the deformed balloon perimeter for the deduction of the BAF value

As the present study is motivated by a concrete application scenario, in the following an exemplary interaction of a simplified balloon configuration in contact with the idealized tissue is discussed (Fig. 11), suggesting simple attempts for quantitative and semi-quantitative analysis. In doing so, the volume and pressure utilized to inflate the balloon are assumed to be known, as with these quantities the vessel evaluation is possible without the need of extra sensor elements inside the balloon. The balloon itself is assumed to have integrated strain sensors able to provide the full circumferential extension (viz. the balloon perimeter, see Fig. 11, position S_Peri) and via finer distributed small sensors the initial contact points and the evolution of the same (Fig. 11, position of strain sensors S_Con1 & S_Con2), so it allows the deduction of the BAF value. Further, the inflation behaviour of the balloon without surrounding tissue is known. As basis for the discussion, the 8-wrinkled tissue with averaged tissue parameters (compliance) is taken, as the pressure levels of the max. BAF value and max. CCF value coincide (Fig. 7c), and the balloon structure is the one with the thinnest balloon wall (max. compliance). As mentioned earlier, this can be considered as ideal case to determine the shape and stiffness of the vessel segment.

**Fig. 12:**
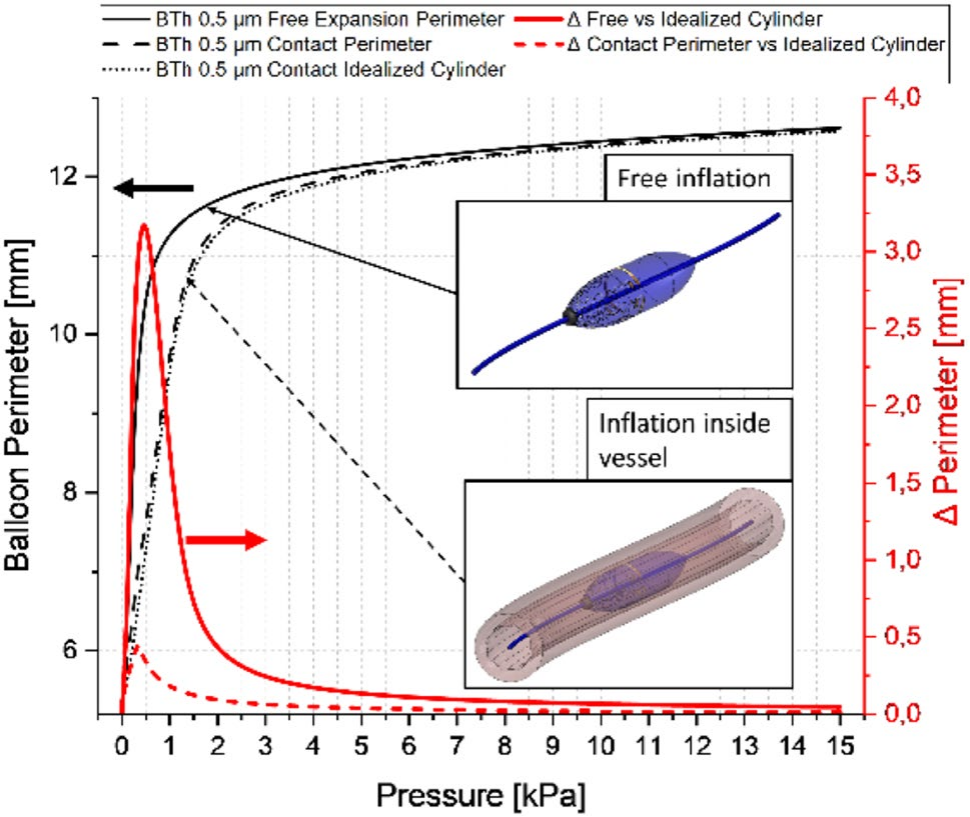
Plot of an inflation sequence of a sensor-balloon with and without surrounding (8 wrinkled) tissue and the calculated cylinder perimeter at the most inner points. Additionally, the differences are shown ( right y-axis). The insets schematically show the two different situations which are compared. The term contact perimeter refers to the deformed balloon perimeter in contact with the wrinkled tissue

**Fig. 13:**
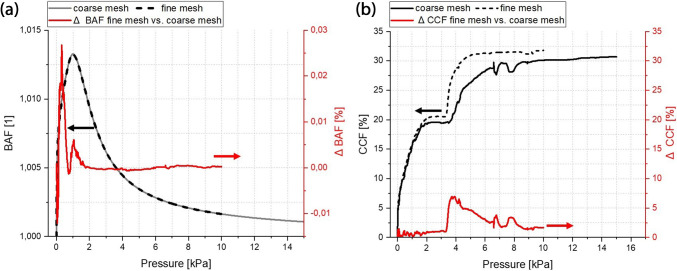
Results of a coarser meshed model compared with a finer meshed model. **a** Superimposed BAF value for the fine meshed model and the coarse meshed reference. The coarse meshed curve is plotted grey to make the dotted fine mesh visible. The right axis refers to the relative difference with respect to the coarse reference mesh. **b** Comparison between CCF value for the fine meshing with the coarse reference. The right axis refers to the relative difference with respect to the coarse reference mesh

**Fig. 14:**
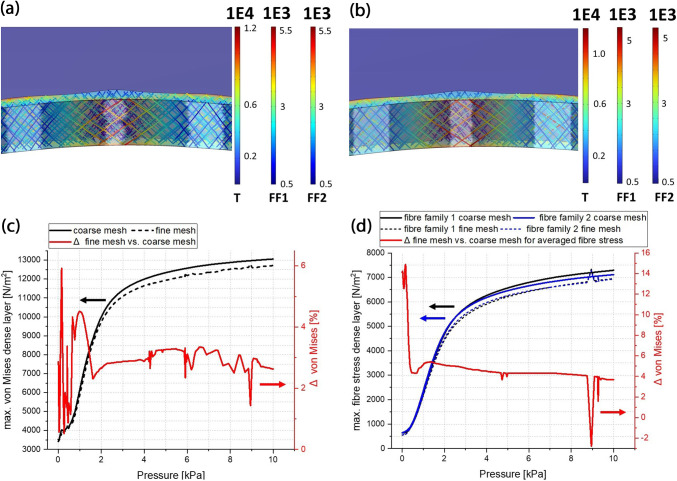
Comparison of the coarse meshed model with the fine meshed model. **a** & **b** von Mises stress distribution path for the coarse **a** and fine **b** meshed model within the dense tissue layer at 7 kPa balloon inflation pressure. **c** Comparison of the max. von Mises stress inside the dense tissue layer up to 10 kPa inflation pressure. The right axis refers to the relative difference with respect to the coarse reference model. **d** Comparison of the max. fibre stress for each family of the coarse and fine meshed model in the dense tissue layer. The right axis refers to the relative difference of the averaged fibre family stress with respect to the coarse reference model

In Fig. 12 the balloon perimeter for an inflation sequence of a sensor balloon is plotted, for a free expansion without surrounding tissue and for an expansion inside a 8-wrinkled vessel segment. Additionally, the cylinder perimeter is plotted, based on the initial contact points. Further the differences between the corresponding curves are shown and related to a second y-axis.

Looking at the asymptotic curve progression (Fig. 12) it is clear that the free reference inflation and the balloon perimeter as well as the idealized cylindrical perimeter are coinciding at high-pressure levels. This is expected as the balloon material is stiffening at larger pressure levels. Taking the obtainable differences into account, the low-pressure regime for such sensing balloons is reassured by that finding.

For the exemplarily taken sensor-balloon tissue interaction (Fig. 11), small strain sensors allow the determination of the initial contact side and the expansion in between two contact points can be treated as thin plate/membrane deflection problem. Thereby, the initial length is given by the current balloon expansion at the initial contact and the initial angle between the two sensor segments defined within the balloon layout. Therefore, the length at the initial contact $l\left(0\right)$ can be described by

14 $$l\left( 0 \right) = 2 \times peri_{fr - cy} \left( 0 \right) \times \frac{\alpha }{360}$$

where ${peri}_{fr-cy}\left(0\right)$, being the calculated cylindrical perimeter at initial contact and $\alpha$ is the defined angle between the two sensor segments measured from the balloon axis.

Adapting the solutions from He et al. (He et al. 2022), the formulation for the maximum thin plate deflection ${\omega }_{0}(i)$ is given by

15 $$\omega_{0} \left( i \right) = \frac{{P\left( i \right) \times l\left( i \right)^{4} \times \left( {1 - \nu^{2} } \right)}}{{2 \times E_{bal} \left( i \right) \times t\left( i \right)^{3} }}$$

or treated as membrane deflection problem

16 $$\omega_{0} \left( i \right) = \sqrt[3]{{\frac{175}{{256}}\frac{{P\left( i \right) \times l\left( i \right)^{4} \times \left( {1 - \nu^{2} } \right)}}{{E_{bal} \left( i \right) \times t\left( i \right)}}}}$$

where $P(i)$ is the current inflation pressure, $l(i)$ and $t(i)$ are the current membrane length and thickness, and ${E}_{bal}(i)$ is the effective Young’s modulus of the balloon including the sensors. Whether the thin plate (19) or the membrane (20) formulation is applicable, mainly depends on the balloon material and the thickness. The results in Fig. 7a suggest a cubic dependence between deflection and thickness, and therefore, the thin plate relation may suite better for a lumped reformulation of the problem.

As for the currently discussed model configuration the pressure level at the max. BAF and max. CCF coincides, the membrane (balloon) deflection at the pressure level where the maximum BAF is reached resembles fully the tissue shape. Looking at adapted Eq. (19) and (20) it becomes clear that the dependence of the thin plate or membrane parameters on the pressure level (i) is detrimental to the prerequisite of rigid fixed edges, as it is common for the analytical treatment of thin plate/membrane deflection problems. Consequently certain assumptions are necessary to solve a selected equation. Therefore, it is reasonable to define suitable ranges in which certain conditions are ‘fixed’ and the deflection is calculated based on a corresponding parameter set (i.e. $$l$$, $$t$$, $${E}_{bal})$$. A concept for the data evaluation to derive the tissue shape and tissue stiffness is provided in Appendix 5.

From the earlier derived semiquantitative relations and the exemplary discussed sensor-balloon tissue interaction some general design guidelines for such high-compliant sensing balloon catheters can be derived:

1. For an effective shape detection, the vessel structure must be able to imprint its shape to the balloon surface and therefore the analysed interaction must take place within low-pressure regimes and the initial deflated balloon diameter should be in the range of the targeted vessel. Consequently, the compliance ratio between the tissue and the balloon determines the interaction sequence and hence the shape detection capability of the balloon.
2. The compliance (wall thickness or material) of the sensing balloon must be able to detect also non-fibrosed tissue segments for an effective vessel mapping, which results in thin balloon walls and low inflation pressures. This general design aspect leads to high complaint balloon catheters which aim for a pure diagnostic functionality and therefore likely do not cause damage within the surrounding tissue. However, it cannot be used for therapeutic purposes due to the low load such a balloon can create on the tissue.
3. The amount of strain sensors on the balloon surface to derive the vessel shape and stiffness must allow a parameter deduction inside the vessel with suitable reformulations of the problem. As this likely leads to lumped reverse modelling of an underdefined problem, either suitable balloon movements or an increased amount of sensors is necessary. Thereby, suitable reformulations of the interactions can be useful to reverse identify shape and stiffness based on localized sensor outputs.
4. The number of sensors, their arrangement and spacing between two sensor segments, in combination with the initial (deflated) balloon diameter relative to the targeted vessel diameter, defines the possible accuracy of a tactile vessel mapping for a single inflation sequence with static sensor and vessel position.
5. In respect of mechanical tissue properties, a lower tissue compliance generally leads to higher induced balloon deformations and consequently also to larger deviations from a free reference balloon inflation. Assuming a fixed balloon compliance, the required sensor sensitivity for shape and stiffness determination therefore increases for higher compliant tissues and an effective quantitative tactile tissue mapping is only obtainable, if either the balloon compliance or the sensor sensitivity is sufficiently high.

### Limitations of the study

The attempt to cover fundamental interactions with idealized tissue models leads to certain limitations regarding the significance of the results. The parametric description of the tissue can be regarded as the most significant deviation from real world correlates as well as the cylindrical assumptions utilized during the parameter fitting. Further, while nonlinearity of the tissue and the balloon material is covered, the interaction assumes frictionless contact and no shear force transfer is included, which may lead to deviating interactions dependent on the friction coefficient. Also, due to the relatively coarse meshing of the tissue used in this study, especially the stress distribution inside the tissue as well as the CCF determination is prone to inaccuracies.

As outcome from the applied symmetry within the vessel structure, the centre of the balloon always coincides with the centre of the vessel lumen, while in reality a non-centred deforming lumen during inflation can occur, especially for strongly non-circular or circumferential partially fibrosed vessels. Also, the presented interpretation of the results is at the current state relatively rough and especially the utilized lumped models must be adapted to suite better to the FEA model as well as possible real-world interactions. Additionally to the intrinsic inaccuracies of analytical correlates describing the interaction, a real sensor layout will not cover the exact position of initial contact sites. Dependent on the utilized lumped models for reverse identification, this may lead to larger inaccuracies of the derived values and shapes, e.g. the initial length for a membrane treatment influences with the power of 4 the calculated maximum deflection.

## Conclusion and outlook

In the present study, two nonlinear state-of-the-art material models for the balloon as well as the tissue were combined with an idealized non-cylindrical geometry, leading to a large dataset as fundament for the presented interdependencies. The proposed control parameters BAF and CCF to describe the interaction phenomena, as well as the analysed interaction sequences, lead to semiquantitative general interdependencies for different compliance ratios between balloon and tissue, as well as different tissue aspect ratios. Based on this, first formulations of design guidelines for tactile (strain) sensing high-compliant balloon catheters were derived.

The present in-silico study represents a first step towards design rules for such sensing balloon catheters which are able to deduce intraoperative patient-specific tissue data. We expect that this fertilizes further research on such high-compliant sensor balloons, where the balloon complexity and associated costs are in a range which is acceptable for the application in standard procedures and consequently will be utilized beyond dedicated study environments.

Coming from the limitations, the future path is paved accordingly. Based on the present work, our group plans to derive refined analytical formulations, which are suitable for reverse identification of the tissue shape and stiffness based on predefined sensor layouts and further defining more specific design guidelines for such sensing balloon catheters. Refined FEA models thereby will serve as in- silico models for validation, and further corresponding experimental layouts together with suitable tissue models will be used to validate them. The process development for an integration of strain sensors inside balloon catheter walls as well as the electromechanical behaviour characterization of specially designed strain sensor elements is ongoing research in our group.

## Electronic supplementary material

Below is the link to the electronic supplementary material.

Supplementary file1 (XLSX 18 KB)

Supplementary file2 (M 1 KB)

Supplementary file3 (M 14 KB)

## Figures and Tables

**Fig. 15:**
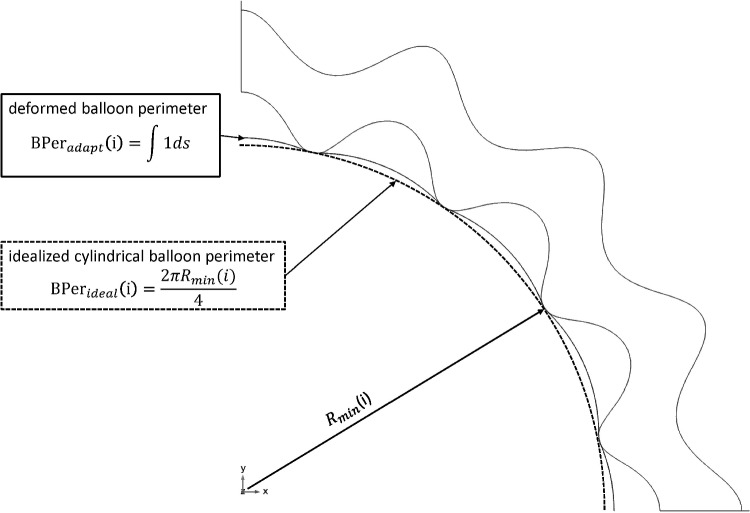
Schematic variable determination for calculation of the balloon compliance-dependent BAF used to characterize the deformation of the balloon during the contact with the tissue

## Data Availability

All calculated data as well as the code files related to parameter identification are available as supplementary files (S1 to S3). Further, the utilized FEA models will be shared on request.

## Appendix 1: Tissue geometry

In a two-dimensional Cartesian coordinate system with the spatial coordinates $x$ and $y$ based on the parameter $s$, the inner lumen curve is given by

17 $$x_{lumen} \left( s \right) = \left[ {meanR + aspectR*\cos \left( {wrinkles \times s} \right)} \right]*{\text{cos}}\left( s \right)$$

and

18 $$y_{lumen} \left( s \right) = \left[ {meanR + aspectR*\cos \left( {wrinkles \times s} \right)} \right]*sin\left( s \right)$$

where $meanR$ represents the mean inner radius of the wrinkled structure (so the circular “zero line” of the inner cosine-function (i.e. $a$ in (8) and (9), defined based on datasets of Cunnane et al. [53]), *aspectR* referring to the depth of the wrinkles (i.e. the amplitude of the inner cosine-function, i.e. *b* in (8) and (9), set to 0.1 for all tissue geometries) and $wrinkles$ referring to the number of wrinkles (based on a full rotation of 2π, i.e. the period of the inner cosine-function, i.e. $d$ in (8) and (9)). To reduce the model size the parameter $s$ runs from $0$ to $\frac{1}{2}$ π. The interface curve between the fibre containing layer and the isotropic surrounding tissue layer is defined by

19 $$x_{interface} \left( s \right) = \left[ {\left( {meanR + urethraTh} \right) + aspectR*\cos \left( {wrinkles \times s} \right)} \right]*{\text{cos}}\left( s \right)$$

and

20 $$y_{interface} \left( s \right) = \left[ {\left( {meanR + urethraTh} \right) + aspectR*\cos \left( {wrinkles \times s} \right)} \right]*{\text{cos}}\left( s \right)$$

where $urethraTh$ is the urethra thickness, based on the Masri et al. (Masri et al. 2018) (i.e. $w$ in (10) and (11)). The outer radius is defined by a simple circular curve, given by

21 $$x_{outerDia} \left( s \right) = outerDia*{\text{cos}}\left( s \right)$$

and

22 $$y_{outerDia} \left( s \right) = outerDia*sin\left( s \right)$$

with $outerDia$ being the outer diameter of the vessel sample (based on datasets of (Cunnane et al. 2021)). The balloon structure is analogously defined, with

23 $$x_{outerDia} \left( s \right) = \left[ {\left( {meanR - aspectR} \right) - distance} \right]*{\text{cos}}\left( s \right)$$

and

24 $$y_{outerDia} \left( s \right) = \left[ {\left( {meanR - aspectR} \right) - distance} \right]*sin\left( s \right)$$

where $distance$ refers to the initial distance between the balloon and the inner wrinkled surface of the tissue, while (23) and (24) define the outer balloon surface. Consequently, the inner balloon surface is described by

25 $$x_{innerDia} \left( s \right) = \left[ {\left( 
{meanR - aspectR} \right) - distance - balloonTh} \right]*{\text{cos}}\left( s \right)$$

and

26 $$y_{innerDia} \left( s \right) = \left[ {\left( {meanR - aspectR} \right) - distance - balloonTh} \right]*sin\left( s \right)$$

where $balloonTh$ constitutes the wall thickness of the balloon and therefore defines the balloon compliance.

## Appendix 2: Parameter identification

For an incompressible tissue wall with the prior mentioned neglection of the residual stresses in circumferential direction, and in respect of a coordinate system with the directions $\theta$, $r$, $z$ representing the circumferential, radial and axial directions, a mapping of the deformed configuration ($r,\theta ,z)$ to the undeformed configuration ($R, \Theta ,Z)$ is given by the corresponding stretch ${\lambda }_{n}$ as

27 $$\lambda_{\theta \theta } = \frac{r}{R}$$

28 $$\lambda_{rr} = \frac{R}{{r\lambda_{zz} }}$$

29 $$\lambda_{zz} = \frac{l}{L}$$

where $L$ and $l$ are the vessel length of the undeformed (ex vivo) and deformed (in-vivo) configuration.

As the invariants ${\overline{I} }_{4}$ and ${\overline{I} }_{6}$ represent the square of the fibre stretches ${\lambda }_{fibre}^{2}$ in a certain direction, ${\lambda }_{fibre}^{2}$ is given by

30 $$\lambda_{fibre}^{2} = {\overline{\text{I}}}_{{4{ }}} = {\overline{\text{I}}}_{6} = {\text{cos}}\left( {{\upbeta }_{0} } \right)^{2} \lambda_{{\theta \theta { }}}^{2} + {\text{sin}}\left( {{\upbeta }_{0} } \right)^{2} \lambda_{zz}^{2}$$

where, as described earlier, the angle $\beta$ thereby is set to 45° based on uniaxial testing in longitudinal and circumferential direction.

As the axial pre-stretch (in-vivo state) ${\lambda }_{zz}$, the average tissue thickness ${t}_{ave}$ and evaluated outer radius ${r}_{ou(0)}$ at 0 input pressure are provided in the datasets of Cunnane et al. (Cunnane et al. 2021), the outer radius ${R}_{ou(0)}$ and the inner radius ${R}_{in(0)}$ in the longitudinal unstretched (ex vivo) state can be computed. In doing so, the assumption that the lumen volume confined within the cylindrical vessel remains constant during axial stretching and further isochoric assumption for the vessel tube lead to

31 $$R_{ou\left( 0 \right)} = r_{ou\left( 0 \right)} {*}\sqrt {\lambda_{zz} }$$

32 $$R_{in\left( 0 \right)} = R_{ou\left( 0 \right)} - t_{ave}$$

Correspondingly, as the outer radius ${r}_{ou\left(i\right)}$ at each pressure level ($i$) is again provided in the datasets of Cunnane et al. (Cunnane et al. 2021), the inner radius ${r}_{in}$ at each pressure level can be computed by

33 $$r_{in\left( 0 \right)} = \frac{{R_{in\left( 0 \right)} }}{{\sqrt {\lambda_{zz} } }}$$

34 $$r_{in\left( i \right)} = \sqrt {r_{ou\left( i \right)}^{2} + r_{in\left( 0 \right)}^{2} - r_{ou\left( 0 \right)}^{2} }$$

Cross-checking the influence of the made lumen volume assumption with FEA results for a cylindrical HGO carotid artery bilayer vessel model (COMSOL AB 1) and a cylindrical tissue model of this study (without wrinkled inner vessel wall) showed a maximum deviation of 5.1% for the inner radius in respect of the exact FEA solution. Based on this, the influence on the identified parameters is rated as not relevant for the current study aim.

As described earlier, based on work from Masri et al. (Masri et al. 2018) and Natali et al. (Natali et al. 2017; Natali et al. 2017), a two-layer tissue model with a denser inner part and a looser outer part is utilized in this study, as this is regarded as state of the art for urethral tissue models. Thereby, Masri et al. (Masri et al. 2018) reported for the denser layer (i.e. the urethra) a thickness of around 0,25 mm. Based on this, the defined bilayer models for the parameter identification consist of denser inner layers with a thickness of 0,25 mm, starting from the lumen in radial direction. This value is kept the same for all datasets as no analysis regarding a varying dense-layer thickness is provided in the utilized datasets of Cunnane et al. (Cunnane et al. 2021). The softer outer layer fills the rest up to the calculated outer radii ${R}_{ou(0)}$ based on Cunnane’s datasets. Consequently, it is necessary to calculate the boundary between the dense (urethral) tissue and the softer surrounding conjunctive tissue layer. Thereby, the boundary radius ${R}_{bou(0)}$ in the undeformed configuration is given by

35 $$R_{bou\left( 0 \right)} = R_{in\left( 0 \right)} + 0.25 mm$$

Correspondingly the boundary radius during deformation ${r}_{bou}$ at each pressure level ($i$) is given by

36 $$r_{bou\left( 0 \right)} = \frac{{R_{bou\left( 0 \right)} }}{{\sqrt {\lambda_{zz} } }}$$

37 $$r_{bou\left( i \right)} = \sqrt {r_{bou\left( 0 \right)}^{2} - r_{in\left( 0 \right)}^{2} + r_{in\left( i \right)}^{2} }$$

For the parameter identification a thick cylindrical wall assumption is used. In doing so, the Cauchy stresses ${\sigma }_{n}$ are determined by:

38 $$\sigma_{n} = - P_{h} + \lambda_{n} \frac{{\partial W_{HGO} }}{{\partial \lambda_{n} }}$$

where ${P}_{h}$ is the hydrostatic pressure, the summed term depends on the stretch and the partial derivative of the SEF w. r. t. the stretch, and $n$ represents the direction of the stretch. An analytical formulation of the circumferential Cauchy stress ${\sigma }_{\theta \theta }$ is given by Eq. (39):

39 $$\sigma_{\theta \theta } = - {\text{P}}_{{\text{h}}} + 2c\lambda_{{\theta \theta { }}}^{2} + 4{\text{k}}_{1} \lambda_{{\theta \theta { }}}^{2} (\lambda_{fibre}^{2} - 1) cos^{2} \left( \beta \right)\left( {{\text{exp}}^{{{\text{k}}_{2} \left( {\lambda_{fibre}^{2} - 1} \right)^{2} }} } \right)$$

and the Cauchy stress in radial direction ${\sigma }_{rr}$ is given by

40 $$\sigma_{rr} = - {\text{P}}_{{\text{h}}} + 2{\text{c}}\lambda_{{rr{ }}}^{2}$$

The boundary conditions ${\sigma }_{rr}\left({r}_{in}\right)=-{P}_{mod}$ and ${\sigma }_{rr}\left({r}_{ou}\right)=0$, assumption of axisymmetry, neglecting torsion, acceleration, body forces and axial extension, lead to the expression for the modelled lumen pressure ${P}_{mod(i)}$

41 $$P_{{{\text{mod}}\left( i \right)}} \left( {c,k_{1} ,k_{2} ,\beta } \right) = \int\limits_{{r_{{{\text{in}}\left( i \right)}} }}^{{r_{{{\text{bo}}u\left( i \right)}} }} {\frac{{\sigma _{{\theta \theta }} - \sigma _{{rr}} }}{r}} dr + \int\limits_{{r_{{{\text{bo}}u\left( i \right)}} }}^{{r_{{{\text{ou}}\left( i \right)}} }} {\frac{{\sigma _{{\theta \theta }} - \sigma _{{rr}} }}{r}} dr$$

where the pressure in Eq. (41) is a function of the parameters $c, {k}_{1}, {k}_{2},\beta$. For each pressure state the deformed inner radius ${r}_{in\left(i\right)}$ of the cylindrical tube is computed based on the provided outer radius ${r}_{ou(i)}$. Further, by substituting Eq. (30) in (39) the circumferential Cauchy stress ${\sigma }_{\theta \theta }$ can be computed and with Eq. (40) the radial Cauchy stress ${\sigma }_{rr}$.

Generally, the reduced axial force is considered as an important aspect for an accurate computation of the material parameters, as it is the additional force in longitudinal direction during pressure inflation testing. As no corresponding information besides the utilized datasets of Cunnane et al. (Cunnane et al. 2021) is available, the reduced axial force is not considered during the optimization process and the axial stretch is held constant by an prescribed displacement.

Further, studies from Natali et al. 2017 on horse urethras, which are regarded similar to human urethras in respect of morphological and biomechanical characteristics, evaluated a ratio for the Cauchy stresses between the denser inner and looser outer layers of around 208 (Natali et al. 2017). Following this, the ratio ($\frac{{c}_{d}}{{c}_{l}}$) for the matrix stiffness parameters ${c}_{d}$ of the dense layer and ${c}_{l}$ of the looser surrounding layer as well as the corresponding ${k}_{1d}$ and ${k}_{1l}$ parameters was set to 208. Together with the preceding boundary conditions for the fibre alignment angle (45°) in the unstretched state, the parameters ${k}_{1}, { k}_{2}, c$ for the dense inner layer (subscript $d$) and the softer outer layer (subscript $l$) can be identified based on the error function (Eq. (42)) with the help of the nonlinear ‘fmincon’ function in MATLAB®

42 $$\text{Error function} = \mathop \sum \limits_{i = 0}^{10} (P_{mod\left( i \right)} \left( {c, k_{1} , k_{2} ,\beta } \right) - P_{input\left( i \right)} )^{2}$$

where ${P}_{input(i)}$ are the pressures within the lumen, considered here from 0 to 10 kPa in 1 kPa steps. The parameters are constraint to positive values. In the ‘fmincon’ MATLAB® function the interior-point approach is used to solve a sequence of approximate minimization problems. This sets components that violate bounds or are equal to a bound to the interior of the bound region. Besides the assumption that no residual stresses are present in the tissue as long as no pre-stretch is applied, the ${k}_{2}$ parameters for the dense and loose layer are set equal. The chosen parameter interdependencies $\frac{{c}_{d}}{{c}_{l}}=208$, $\frac{{k}_{1d}}{{k}_{1l}}=208$ and ${k}_{2d}={k}_{2d}={k}_{2}$ reduce the free parameters from 6 to 3.

It is important to note that this inherently implies a physical interpretation of the parameters, where $c$ represents the corresponding stiffness of the isotropic matrix surrounding the collagen fibres in the dense and loose tissue, while in the loose tissue the porosity is included and lowers the respective values as a continuum is assumed. Further the ${k}_{1}$ parameter can be seen as a density factor for the collagen fibres in the respective tissue layer and therefore resembles a substitute spring force for the collagen fibres in each layer (based on the common single spring interpretation of a single collagen fibre). Utilizing again the ratio of 208 found by Natali et al. (Natali et al. 2017) for the isotropic matrix parameter and the collagen stiffness parameter implies that the ground substance and also the relative amount of collagen fibres is lower in the looser surrounding tissue. This trend qualitatively correlates to histological cuts (Natali et al. 2017). Lastly, keeping ${k}_{2}$ equal for each layer, it can be interpreted as the stiffening behaviour of the collagen fibres being the same for each tissue layer, implying that the equilibrium state of an unloaded collagen fibre is always the same.

Cunnane’s et al. dataset (Cunnane et al. 2021) provides 9 samples which were each analysed for pressures within the lumen from 0 to 10 kPa. The parameter identification procedure was done individually for 8 samples, while sample 4 was left out as the isochoric assumption with the provided average thickness leads to a negative inner radius in the undeformed state (which can be attributed to the only roughly derived average thickness calculation utilized by Cunnane et al. (Cunnane et al. 2021)). Looking at the identified parameters (Table 2) it is obvious that for certain samples the parameter identification leads to values, which make physically no sense as they stand against the physical interpretation described above. The high variance within the identified values (Table 2) can be directly attributed to the utilized cylindrical thick wall assumption during the parameter identification, as the real lumen shape is deviating largely from an idealized cylindrical form. As Cunnane et al. (Cunnane et al. 2021) provide the extracted lumen shapes for each sample, it can be seen that especially for strongly branched lumen shapes (e.g. sample 8 and 9) the stiffness values for the isotropic matrix are extremely low. Thereby, the geometrical unfolding during initial pressurization leads to an extreme high compliance for the matrix stiffness values as due to the cylindrical assumption the deformation is directly attributed to the material instead of geometrical attributes.

**Table 2:** Identified parameters for each sample from Cunnane et al. dataset (Cunnane et al. 2021) and the utilized averaged values for the FEM model. For the average values, only samples indicated by * were considered. Parameters of sample no. 1 were taken for BAF and CCF comparison with a stiffer tissue segment

Sample No	${c}_{d}$[kPa] dense layer	${k}_{1d}$[kPa] dense layer	${c}_{l}$[kPa] loose layer	${k}_{1l}$[kPa] loose layer	${k}_{2}$ dense/loose layer
1*	6.1164	2.7763	2.9406e–02	1.3348e–02	0.1495
2*	1.5522	0.20	7.4625e–03	9.6154e–04	1.0e–03
3*	3.5570	1.6110	1.710e-02	7.7452e–03	5.690e–02
5*	0.4450	4.290	2.1394e–03	2.0625e–02	4.1550e–02
6*	0.3554	0.1126	1.7087e–03	5.4135e–04	1.350e–04
7*	9.4147	0.5899	4.5263e–02	2.8361e–03	0.7774
8	0.0001	10.5758	4.8077e–07	5.0845e–02	0.13656
9	0.0003	7.5280e–02	1.4423e–06	3.6192e–04	1.090e–03
ave.* (for FEM model)	3.5735	1.5966	1.7180e-02	7.6762e-03	0.1711

As a consequence, sample 8 and 9 were excluded and the utilized base geometry (inner mean radius, outer mean radius) as well as the selected parameters of the FEM model are based on the averaged values of sample 1 to 7 (6 in total, as 4 is excluded). The full data calculated based on Cunnane’s dataset as well as the corresponding MATLAB® code are provided in the supplementary data (Online Resource S1, Online Resource S2, Online Resource S3).

## Appendix 3: Model meshing

An effective way to implement a mesh refinement within COMSOL® is an adaptive mesh refinement study. Thereby, the solution is computed based on an initial mesh while evaluating the residuals of the partial-differential equation (PDE) for all mesh elements. In case the estimated error of the mesh element solutions is getting to high, the geometry will be re-meshed with finer elements in the corresponding regions based on the sizes of the local error indicators (L_2_ norm of the gradient of the dependent variable) and the time integration is restarted (COMSOL AB 2). As starting point for the meshing the BTh 0.5 with the 12 wrinkled tissue model was chosen, as it was expected that this setup with intermediate aspect ratio and maximum balloon compliance is a good representative for all other model configurations, in respect of occurring deformation states. As starting mesh for the balloon structure a free triangular surface mesh which is extruded along the radial direction is chosen and the maximum element size was set to 0.03 mm. The tissue is meshed with free tetrahedral elements, while the build in size control of COMSOL® is used. Thereby, the size control is calibrated to the general physics (viz. the mesh size and size distribution are chosen based on the solid mechanics interface) and the denser tissue layer is meshed finer than the looser outer layer. This starting mesh configuration was transferred to every other model setup (leading to a differing element count in the varying tissue geometries) and a simulation run was started. If the run was not converging up to the chosen limit of 15 kPa inflation pressure, either the balloon mesh or the dense tissue layer mesh was refined. The refinement by adapting the size control setups for the corresponding layers was done till a converging run up to 15 kPa was achieved for each balloon tissue combination. This iterative mesh refinement procedure leads to deviating starting meshes for differing and also for equal tissue geometries, which is influencing the comparability of the results. However, the resulting deviation in number of mesh elements is regarded as not relevant for the conducted trend analysis within this study. To justify this viewpoint, a highly refined mesh (≈ 12 × finer mesh in the dense tissue layer and ≈ 9 × finer mesh in the balloon structure) for the combination of BTh 1.5 with the 12 wrinkled tissue model was simulated up to 10 kPa. Comparing the coarser meshed model with the refined meshing (Fig.13a + b) it is visible that the obtained trend is not changed, while it becomes clear that especially for the CCF parameter inaccuracies are directly related to coarser mesh elements as this leads to an inaccurate determination of the in-contact variable (1 or 0) within COMSOL®. While the maximum error is around 7.5% the trend which is relevant for the conducted analysis is not changed. Further it is visible that the noise associated with inaccuracies of the in-contact variable utilized to obtain the CCF value is reduced as the resolution of contacting mesh nodes is increased (Fig. 13b).

In Fig. 14a, b a comparison between the fine and coarse mesh model for the volume and fibre stress distribution as well as the contact surface is shown. Thereby it can be seen that the stress distribution path in opposing manner to the tissue geometry is not changed, while the contact area is refined. Figure S14c shows the max. von Mises stress inside the isotropic matrix of the dense tissue layer for the coarse and fine meshed model, while the maximum difference is around 6% in the low-pressure region. Figure S14d displays the max. fibre stresses for each fibre family for the fine and coarse meshed model and the difference for the averaged fibre stresses of each mesh setup. It can be seen that in general the max. stress values in the fine meshed model are lower (except the inaccuracy at around 9 kPa for the fine meshed model Fig. 14d). This holds true for the volume stress in the isotropic matrix as well as the fibre stress. Furthermore, in Fig. 14d it can be seen that the fibre stress in the coarse meshed model differs between the fibre families which can be directly attributed to the coarse meshing as the starting angle between the fibre families is 45°. For the fine meshed model this difference decreases. The unreasonable high stress peak at around 9 kPa within the fibre stresses is slightly also observable in the isotropic matrix and is likely associated with local mesh element distortion at higher deformation levels.

The corresponding starting and final adapted mesh data can be seen in Table 3. Thereby, the number of elements and the average element quality is shown, while the element quality is related to the skewness of an element. This is the default element quality measure within COMSOL® and relates the regularity of a mesh element to its equiangular skew viz. a value of 1 is a perfectly regular element and 0 represents a degenerated element (COMSOL AB 2).

**Table 3:** Mesh data for each simulation model. The starting meshes correspond to the meshes which lead to an converging run up to 15 kPa inflation pressure and are an outcome of an iterative mesh refinement of the balloon and/or the dense tissue layer based on the transferred configuration from BTh 0.5 + 12 wrinkled tissue. The final adapted mesh corresponds to the adaptive mesh refinement conducted for each simulation run and was limited to maximum 8 iterations

BTh [µm] / tissue wrinkles [no. per 360°]	Starting mesh for balloon / dense tissue layer / loose tissue layer [element no. (average element quality)]	Fnal adapted mesh for balloon / dense tissue layer / loose tissue layer [element no. (average element quality)]
0.5 / 8	2040 (0.92) / 415 (0.67) / 1263 (0.53)	2040 (0.92) / 927 (0.60) / 8772 (0.52)
1.0 / 8	922 ( 0.94) / 679 (0.67) / 1431 (0.55)	922 (0.94) / 700 (0.67) / 8224 (0.53)
1.5 / 8	922 (0.91) / 1312 (0.66) /1594 (058)	922 (0.91) / 1312 (0.66) / 8976 (0.56)
2.0 / 8	2040 (0.92) / 415 (0.67) / 1263 (0.53)	2040 (0.92) / 909 (0.60) / 8535 (0.52)
2.5 / 8	920 (0.91) / 679 (0.67) / 1332 (0.57)	920 (0.91) / 699 (0.66) / 8181 (0.55)
0.5 / 12	922 (0.93) / 1979 (0.65) / 1804 (0.60)	922 (0.66) / 1979 (0.65) / 10,102 (0.56)
1.0 / 12	922 (0.91) / 2239 (0.66) / 2322 (0.60)	922 (0.91) / 2239 (0.66) / 11,155 (0.57)
1.5 / 12	922 (0.93) / 1979 (0.65) / 1804 (0.60)	922 (0.93) / 1979 (0.65) / 9988 (0.56)
1.5 / 12 fine	8094 ( 0.95) / 24,224 (0.67) / 7891 (0.66)	8094 (0.95) / 24,224 (0.67) / 100,887 (0.62)
2.0 / 12	922 (0.93) /1984 (0.65) / 1787 (0.61)	922 (0.93) / 1984 (0.65) / 9831 (0.57)
2.5 / 12	922 (0.93) / 1984 (0.65) / 1787 (0.61)	922 (0.93) / 1984 (0.65) / 9920 (0.57)
0.5 / 16	2038 (0.92) / 3385 (0.66) / 2998 (0.63)	2038 (0.92) / 3385 (0.66) / 16,833 (0.58)
1.0 / 16	922 (0.94) / 4899 (0.65) / 4383 (0.63)	922 (0.94) / 4899 (0.65) / 23,536 (0.58)
1.5 / 16	922 (0.94) / 4865 (0.66) / 4371 (0.62)	922 (0.94) / 4865 (0.66) / 22,274 (0.57)
2.0 / 16	922 (0.94) / 4865 (0.66) / 4371 (0.62)	922 (0.94) / 4865 (0.66) / 21,041 (0.57)
2.5 / 16	2040 (0.92) / 3370 (0.66) / 2948 (0.62)	2040 (0.92) / 3370 (0.66) / 15,737 (0.57)

Concluding the exemplary mesh refinement study it is clear that for the conducted semiquantitative analysis in this work the relative coarse meshing is not interfering with the conclusions drawn and proves the validity of the obtained results.

However, aiming at more detailed quantitative analysis for specific interaction sequences, special care has to be taken with regard to meshing as the generally challenging analysis of a contact problem with two nonlinear high-compliant materials needs sufficiently fine meshing in order to avoid inaccuracies associated with element distortion.

## Appendix 4: Determination of BAF

In Fig. 15 the variable determination for the balloon adaption factor (BAF) is schematically shown. Thereby the most inner contact point with the tissue determines the minimal radius ${R}_{min}(i)$ as

43 $$R_{min} \left( i \right) = R_{initial} + u_{min} \left( i \right)$$

where ${R}_{initial}$ is the initial balloon diameter, accordingly to equations 23/ 24 given by

44 $$R_{intial} = \left( {meanR - aspectR} \right) - distance$$

and ${u}_{min}$(i) represents the minimal displacement of the outer balloon edge along the integration path of ${BPer}_{adapt}(i)$ viz. the outer surface edge of the balloon in contact with the tissue.

## Tissue stiffness

Aiming at analysing the tissue stiffness, the evolution of the most inner contact points, viz. the idealized cylindrical perimeter determined with circumferentially distributed small sensor elements, can be related to the compression of the tissue wrinkles orientated towards the lumen side. Subtracting the free inflation perimeter from the calculated cylindrical perimeter thereby leads to a difference in compression which is dependent on the tissue stiffness. As the pressure range is the same for the inflation sequence, a force-compression relation can be derived which can be used to characterize the tissue’s Young’s modulus of the isotropic matrix and at higher pressure levels the stiffening of the tissue due to the collagen fibre stretching. As soon as the balloon comes in contact with the tissue, the cylindrical perimeter curve is deviating from the free perimeter curve, and certain small sensor elements of the sensor array are in contact with the vessel wrinkles (Fig. 11). From here on, the effective stiffness of the corresponding sensor element is an addition of the balloon and tissue wrinkle stiffness.

Assuming a linear elastic balloon deformation and an isotropic linear tissue compression within the low-pressure range, for a symmetrically wrinkled vessel structure the following relations can be deduced:

45 $$E_{{{\text{eff}}}} \left( i \right) = E_{{{\text{bal}}}} \left( i \right) + E_{{{\text{tiss}}}} \left( i \right) = \frac{{P\left( i \right) - P\left( 0 \right)}}{{\frac{{\left( {{\text{peri}}_{{{\text{fr}}}} \left( i \right) - \Delta {\text{peri}}_{{{\text{fr}} - {\text{cy}}}} \left( i \right)} \right) - {\text{peri}}_{{{\text{fr}}}} \left( 0 \right)}}{{{\text{peri}}_{{{\text{fr}}}} \left( 0 \right)}}}};i \le i_{{{\text{nonlinear}}}}$$

where ${E}_{eff}$ is the Young’s modulus of the balloon in contact with tissue, ${E}_{bal}$ is the Young’s modulus of the balloon including the sensors and ${E}_{tiss}$ of the tissue, respectively, $P\left(i\right)\, and\, P(0)$ are the inflation pressures at the current state and at the contact point, respectively, ${peri}_{fr}(i)$ is the corresponding perimeter of the free balloon inflation, ${\Delta peri}_{fr-cy}(i)$ the current difference between the calculated cylindrical perimeter and the free inflation perimeter and ${peri}_{fr}(0)$ the perimeter of the balloon at the contact point, while ${i}_{nonlinear}$ represents the pressure level at which the linear assumption is no longer applicable. ${E}_{bal}\left(i\right)$ is given by Eq. (46):

46 $$E_{{{\text{bal}}}} \left( i \right) = \frac{P\left( i \right)}{{\frac{{\left( {{\text{peri}}_{{{\text{fr}}}} \left( i \right) - {\text{peri}}_{{{\text{fr}}}} \left( 0 \right)} \right)}}{{{\text{peri}}_{fr} \left( 0 \right)}}}}$$

and replacing ${peri}_{fr}\left(i\right)-{\Delta peri}_{fr-cy}\left(i\right)$ with ${peri}_{fr-cy}\left(i\right)$, being the current calculated cylindrical perimeter, rearranging of Eq. (45) leads to

47 $$E_{{{\text{tiss}}}} \left( i \right) = \frac{P\left( i \right) - P\left( 0 \right)}{{\frac{{{\text{peri}}_{{{\text{fr}} - {\text{cy}}}} \left( i \right) - {\text{peri}}_{{{\text{fr}}}} \left( 0 \right)}}{{{\text{peri}}_{{{\text{fr}}}} \left( 0 \right)}}}} - \frac{P\left( i \right)}{{\frac{{{\text{peri}}_{{{\text{fr}}}} \left( i \right) - {\text{peri}}_{{{\text{fr}}}} \left( 0 \right)}}{{{\text{peri}}_{{{\text{fr}}}} \left( 0 \right)}}}}$$

and with the simplification $P\left(0\right)$ = 0, it can be rewritten as

48 $$E_{{{\text{tiss}}}} \left( i \right) = \frac{P\left( i \right)}{{\frac{{{\text{peri}}_{{{\text{fr}} - {\text{cy}}}} \left( i \right) - {\text{peri}}_{{{\text{fr}}}} \left( 0 \right)}}{{{\text{peri}}_{{{\text{fr}}}} \left( 0 \right)}}}} - \frac{P\left( i \right)}{{\frac{{{\text{peri}}_{{{\text{fr}}}} \left( i 
\right) - {\text{peri}}_{{{\text{fr}}}} \left( 0 \right)}}{{{\text{peri}}_{{{\text{fr}}}} \left( 0 \right)}}}}$$

## Tissue shape

To evaluate the shape of the tissue, the BAF parameter is a meaningful quantity, as it allows directly a semiquantitative assessment of the vessel shape, as larger values correspond to larger deviations from the calculated inner circle and consequently larger vessel deviations from a cylindrical reference (for the high-compliant inflation regime). While in the present work the value BAF always relates the current contact perimeter to a circular path along the inner contact points, for real vessel structures (especially urethral tissue) it is reasonable to include also elliptical paths along inner points as this can account for certain vessel eccentricity. With the additional circumferentially segmented sensors this allows for example in blood vessels directly a relation of the BAF value to the circumferential extension of plaques and the corresponding lumen occlusion.

For the exemplary balloon tissue interaction (Fig. 11), the ‘final’ thin plate /membrane state of interest is reached at the maximum BAF. In an attempt to keep it simple, a possible approach is to calculate the maximum deflection based on an initial contact state, with the thin plate/membrane length according to Eq. (14). As the tissue is constantly compressed during the subsequent pressurization, the calculated deflection must then be corrected by a factor which considers the increasing membrane length due to radial expansion of the contact sides and the compression of the tissue at the same. Taking Eq. (15) for the maximum thin plate deflection, a possible approach to calculate the wrinkle depth would be

49 $$\omega_{0} \left( i \right) = \left( {\frac{{P\left( i \right) \times l\left( 0 \right)^{4} \times \left( {1 - \nu^{2} } \right)}}{{2 \times E_{{{\text{bal}}}} \left( 0 \right) \times t\left( 0 \right)^{3} }}} \right) {\text{CF}}$$

where ${E}_{bal}\left(0\right), l\left(0\right)$ and $t\left(0\right)$ are the Young’s modulus, length and thickness at the initial contact state and $P\left(i\right)$ is the pressure delta between the initial contact state and the pressure level where the maximum BAF is reached. $CF$ is the correction factor accounting for the compression and radial expansion of the contact sites.

Exemplarily, this represents a simple and first step for a possible lumped model, while the determination of the correction factor $CF$ needs FEA-based studies on the relevant interaction sequences or a refined analytical formulation must be used. Further, the ‘initial’ thin plate/membrane state cannot be regarded as stress free and a term considering residual stresses must be introduced. Also, dependent on the aspect ratio of the wrinkles, the evolving tissue contact must be taken into account.
