# Correction: Finite element analysis of the interaction between high-compliant balloon catheters and non-cylindrical vessel structures: towards tactile sensing balloon catheters

**DOI:** 10.1007/s10237-025-02017-7

**Published:** 2025-10-27

**Authors:** Ashish Bhave, Benjamin Sittkus, Gerald Urban, Ulrich Mescheder, Knut Möller

**Affiliations:** 1 Institute of Technical Medicine (ITeM), Furtwangen University, 78054 Villingen‑Schwenningen, Germany; 2 Department of Microsystems Engineering, IMTEK, University of Freiburg, 79110 Freiburg, Germany; 3 Institute for Microsystems Technology (iMST), Furtwangen University, 78120 Furtwangen, Germany; 4 Associated to the Faculty of Engineering, University of Freiburg, 79110 Freiburg, Germany; 5 Department of Mechanical Engineering, University of Canterbury, Christchurch, New Zealand

**Correction to: Biomechanics and Modeling in Mechanobiology (2023) 22:2033–2061** 10.1007/s10237-023-01749-8

In the original version of the article, the wrong figures appeared as Fig. 3. and Fig. 12; the figures should have appeared as shown below

Incorrect version:

**Fig. 3:**
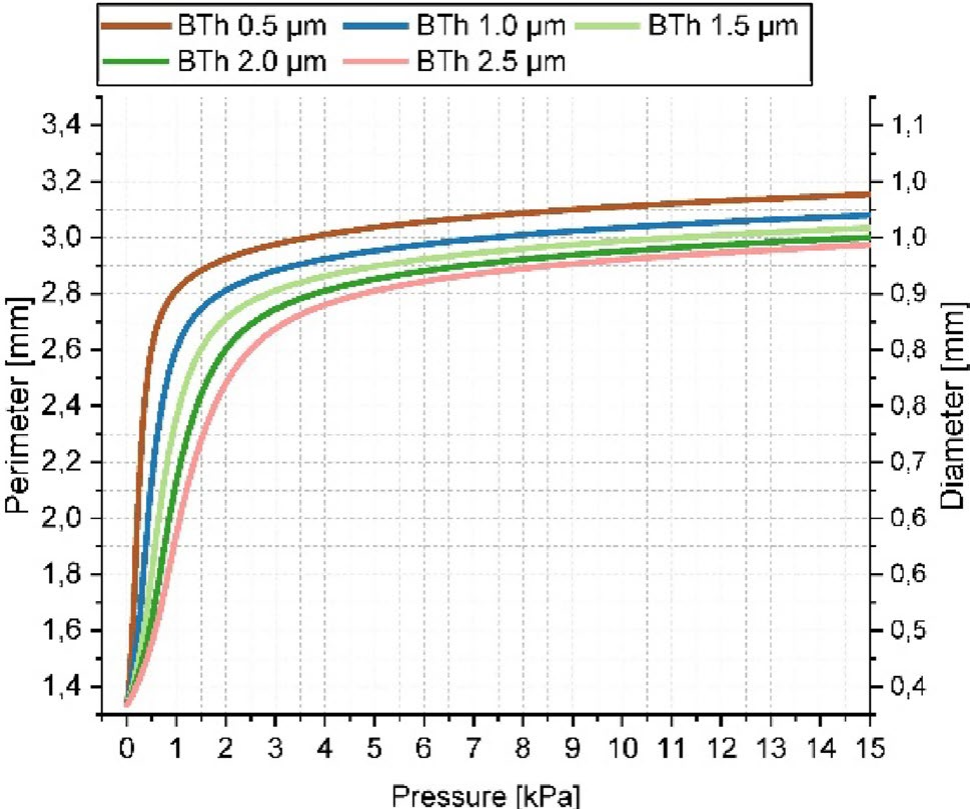
Simulated inflation up to 15 kPa of circular balloon structures with 5 varying PDMS thickness values between 0.5 and 2.5 μm in 0.5 μm steps

Correct version:

**Fig. 3:**
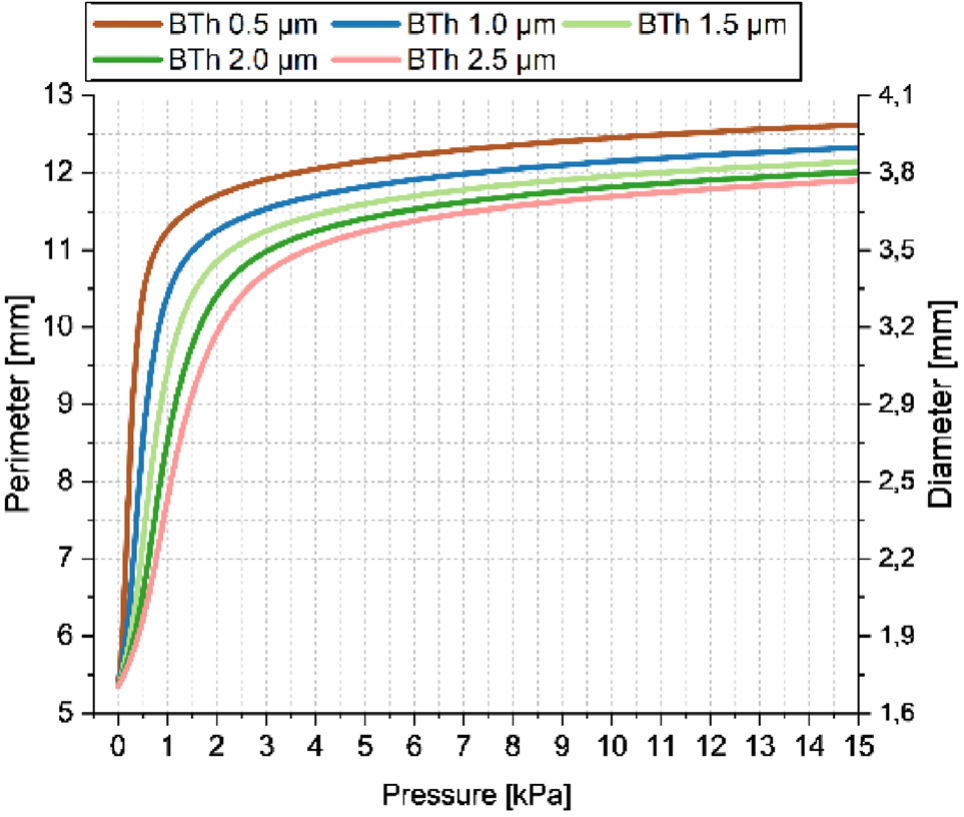
Simulated inflation up to 15 kPa of circular balloon structures with 5 varying PDMS thickness values between 0.5 and 2.5 μm in 0.5 μm steps

Incorrect version:

**Fig. 12:**
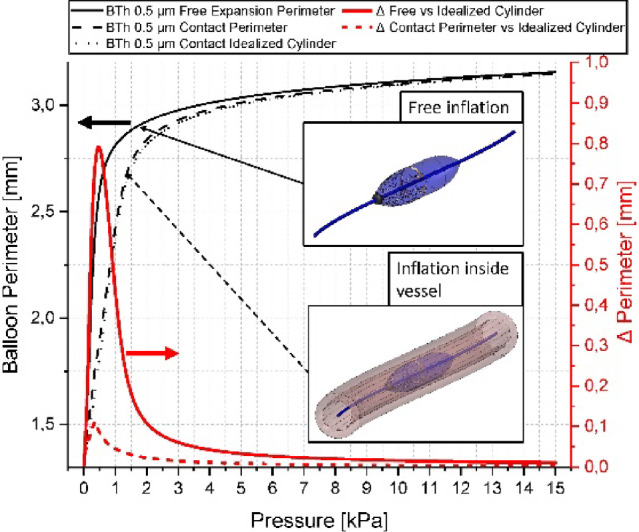
Plot of an inflation sequence of a sensor-balloon with and without surrounding (8 wrinkled) tissue and the calculated cylinder perimeter at the most inner points. Additionally, the differences are shown (right y-axis). The insets schematically show the two different situations which are compared. The term contact perimeter refers to the deformed balloon perimeter in contact with the wrinkled tissue

Correct version:

**Fig. 12:**
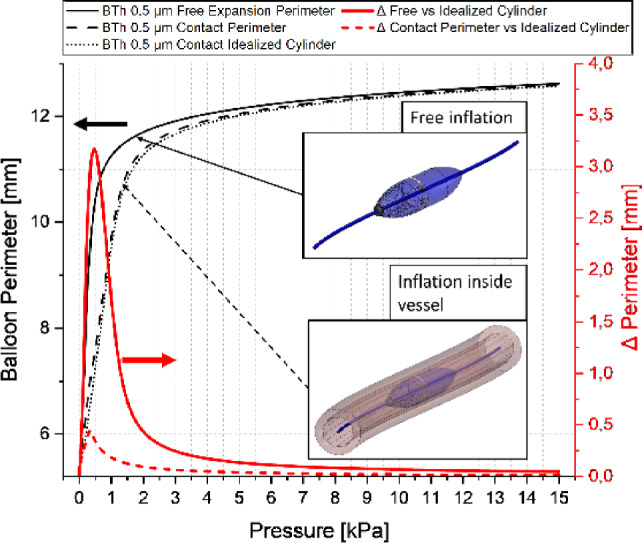
Plot of an inflation sequence of a sensor-balloon with and without surrounding (8 wrinkled) tissue and the calculated cylinder perimeter at the most inner points. Additionally, the differences are shown (right y-axis). The insets schematically show the two different situations which are compared. The term contact perimeter refers to the deformed balloon perimeter in contact with the wrinkled tissue

In Eq. 12, after the variable “CA(i)” in the numerator part a bracket is missing and the formula should have been

Incorrect version:

12 $$CCF\left( {P_{input} \left( i \right),BTh} \right) = \left( {1 - \frac{(BA\left( i \right) - CA\left( i \right)}{{BA\left( i \right)}}} \right)$$

Correct version:

12 $$CCF\left( {P_{input} \left( i \right),BTh} \right) = 1 - \frac{{\left( {BA\left( i \right) - CA\left( i \right)} \right)}}{BA\left( i \right)}$$

In Appendix 1: Tissue geometry section, information about an utilized value was not given as

aspectR referring to the depth of the wrinkles (i.e. the amplitude of the inner cosine-function, i.e. b in (8) and (9))

and should have been

aspectR referring to the depth of the wrinkles (i.e. the amplitude of the inner cosine-function, i.e. b in (8) and (9), set to 0.1 for all tissue geometries)

In Appendix 3: Model meshing section, the citation information was incorrectly given as

Further it is visible that the noise associated with inaccuracies of the in-contact variable utilized to obtain the CCF value is reduced as the resolution of contacting mesh nodes is increased (Fig. 12b)

and should have been

Further it is visible that the noise associated with inaccuracies of the in-contact variable utilized to obtain the CCF value is reduced as the resolution of contacting mesh nodes is increased (Fig. 13b)

In equations 23, 24, 25, 26 and 44 the variable name “aspectR” was termed wrongly as “aspectRa”.

